# Context-dependent toxicity of human tau isoforms in a *Drosophila* tauopathy model

**DOI:** 10.1242/bio.062644

**Published:** 2026-07-28

**Authors:** Yelena Ivanova, Miguel Ramirez-Moreno, Jie Liu, Leila Abtahi, Baidong Wu, Amber S. Cooper, Zengbo Wang, Douglas W. Allan, Amritpal Mudher, Aaron A. Comeault, Lovesha Sivanatharajah

**Affiliations:** ^1^ School of Environmental and Natural Sciences, Bangor University, Bangor LL57 2UR, UK; ^2^ Centre for Biological Sciences, University of Southampton, Southampton SO17 1BJ, UK; ^3^ Department of Cellular and Physiological Sciences, University of British Columbia, Vancouver V6T 1Z3, Canada; ^4^ School of Computer Sciences and Engineering, Bangor University, Bangor LL57 1UT, UK

**Keywords:** Tauopathy, Fly model, Tau isoforms, Selective vulnerability, Tissue selectivity

## Abstract

Tauopathies are characterised by progressive deterioration of brain regions due to abnormal accumulation of microtubule-associated protein tau (MAPT). Alternative splicing of MAPT pre-mRNA produces six isoforms, classified as 3R or 4R depending on whether they contain three or four microtubule-binding repeats. Although many tauopathies are 3R or 4R specific, individual isoform contributions to neurotoxicity remain unclear. To investigate isoform-specific toxicity, we generated *Drosophila* lines with transgenes encoding each human tau (hTau) isoform inserted at the same locus. In young adults, hTau abundance did not differ significantly among lines, yet expression levels were sufficient to induce visible, isoform-specific phenotypes. Across assays, hTau toxicity depended on expression window, tissue type, and neuronal identity; 4R isoforms were generally more toxic than 3R isoforms, but individual isoform effects differed by context. In selected neuronal populations, neurons classed as vulnerable showed early degeneration and continued decline, whereas resilient neurons degenerated only at the later time point. This suggests that, in some neurons, resilience may reflect early resistance to disease processes that is lost with age and exposure to age-related stressors. These phenotypes were not readily explained by hTau abundance or AT8-positive hTau. Together, we show that tau toxicity emerges from interactions between isoform-specific properties and cellular environment.

## Teaser

**Summary:** Human tau isoforms cause distinct patterns of toxicity in *Drosophila*, shaped by tissue type, neuronal identity, and expression timing.

## INTRODUCTION

Microtubule-associated protein tau (MAPT) is a neuronally expressed protein that plays an important role in maintaining neuronal stability and facilitating axonal transport (Barbier et al., 2019). Abnormal tau accumulation is characteristic of several neurodegenerative diseases, including Alzheimer's disease (AD), that are collectively termed tauopathies. In these diseases, tau becomes hyperphosphorylated, which reduces its affinity for microtubules and leads to cytoskeletal destabilisation (Grundke-Iqbal et al., 1986). Pathological tau forms oligomers and larger aggregates [e.g. neurofibrillary tangles (NFTs)], which can disrupt normal cellular transport and synaptic function, ultimately contributing to neuronal degeneration and death (Ballatore et al., 2007).

In the adult human brain, there are six major tau isoforms, all arising from the alternative splicing of the MAPT pre-mRNA (Goedert et al., 1989). Exons 2, 3, and 10 are alternatively spliced, with exon 3 never appearing independently of exon 2. This translates to differences in the number of N-terminal motifs (0-2N, exons 2 and 3) and microtubule-binding repeats (3-4R, exon 10). The smallest tau isoform, 0N3R, is 48 kDa, whilst the largest isoform, 2N4R, is 67 kDa. Depending on exon 10 inclusion in the mRNA, the isoforms can be divided into 3R and 4R groups. During development, tau expression is restricted to 3R isoforms, with 0N3R being the only isoform found in the foetal brain, whereas both 3R and 4R hTau isoforms are expressed in the adult brain (Goedert and Jakes, 1990; Goedert et al., 1989).

In healthy adult brains, the levels of 3R and 4R isoforms are approximately equal, and an imbalance in the 3R:4R ratio is associated with disease (Andreadis, 2005). In AD, both 3R and 4R isoforms are found within NFTs (Goedert et al., 1989), but many tauopathies are 3R or 4R specific, with different types of tau isoforms prevalent in the aggregates. In Pick's disease, there is a predominance of 3R tau, while in progressive supranuclear palsy (PSP), corticobasal degeneration (CBD), argyrophilic grain disease, and globular glial tauopathy, there is a predominance of 4R tau (Falcon et al., 2018; Martinez-Maldonado et al., 2021). These disorders also differ in the cellular and regional distribution of pathology, as well as in their clinical presentation, supporting the idea that specific neuronal populations are selectively vulnerable to distinct tau isoforms (Chung et al., 2021). This suggests that the unique molecular environment of a neuron type may shape its vulnerability to either 3R or 4R species, ultimately contributing to the divergent neuropathological landscapes observed across these diseases.

To date, *Drosophila* disease models have offered a fast and highly translatable experimental platform extensively used to study human tau (hTau) isoforms and their effects on neurodegeneration (reviewed in Sivanantharajah et al., 2019; Varte et al., 2023). Comparisons of the hTau isoforms 0N3R and 0N4R in *Drosophila* have found differences in isoform toxicity (Sealey et al., 2017). Where 0N3R expression was shown to result in axonal transport defects, locomotor impairments, and shorter lifespan, 0N4R expression led to more severe neurodegeneration and memory impairment. A separate study further demonstrated differences in neurotoxicity between the 3R and 4R isoforms (Malmanche et al., 2017). 0N4R and 2N4R expression induced a rough eye phenotype indicative of neurodegeneration, whereas 0N3R did not. Furthermore, this study suggested that developmental expression of 4R hTau was required for the emergence of adult phenotypes like photoreceptor loss and reduced fly lifespan (Malmanche et al., 2017).

Collectively, these studies highlight differences in the neurotoxic effects of 3R and 4R hTau isoforms in *Drosophila*, but the contributions of individual isoforms to tau pathology remain understudied. In particular, 1N3R and 1N4R isoforms are often excluded from the analysis of tau effects on neurodegeneration, limiting our understanding of the full profile of tau toxicity. Furthermore, until recently, many *Drosophila* studies have not systematically accounted for genetic variability like the one arising from the genomic insertion site of transgenic tools (reviewed in Sivanantharajah et al., 2019). Transgenes are subject to position-effect variegation, which can alter expression levels and complicate direct comparisons between isoforms. For studies aimed at understanding isoform-specific tau toxicity, it is therefore imperative to use models with comparable expression of all isoforms being assessed.

Previously, Fernius et al. (2017) created a set of transgenic fly lines in which the six hTau isoforms were inserted into the same genomic locus, VK00018, to enable direct comparison of isoform-specific effects. Analyses of fly survival and behaviour found an overall similar isoform toxicity, with 2N3R expression leading to a significantly reduced lifespan compared to 2N4R. Further investigation of these hTau lines in another study found similar effects on oxidative stress, locomotor behaviour, and survival (Vourkou et al., 2022). In both studies, the effects of the six hTau isoforms on the phenotypes assayed were very mild. One possible explanation is that the hTau expression levels in these lines did not reach the threshold required to induce distinct degenerative phenotypes.

To account for all these challenges, we generated a new set of *Drosophila* lines showing robust expression of hTau isoforms. The six UAS-hTau constructs were inserted into the same landing site, VK00037; but, in contrast to Fernius et al. (2017), 10xUAS constructs were used to greatly boost expression of all hTau isoforms. Examination of these new lines for classic tau-dependent phenotypes revealed clear differences in isoform toxicity. Across all examined tissues, we observed a general trend in which 4R isoforms showed greater toxicity than 3R isoforms. However, the precise hierarchy of isoform-induced toxicity differed between the tissues, indicating that tau-induced degeneration is highly tissue-dependent and shaped by the interactions between specific isoforms and the cell type in which they are expressed, including its unique local molecular landscape. Examination of neurons previously determined to be vulnerable or resilient to hTau0N3R toxicity (Sivanantharajah et al., 2025) revealed differences in the onset and progression of degeneration, with neurons classed as resilient exhibiting degeneration at later stages. This suggests that resilience to tau-mediated degeneration can be age-dependent and context-specific, rather than absolute.

## RESULTS

### New *Drosophila* transgenic lines enable matched comparison of hTau isoforms

**Fig. 1.**
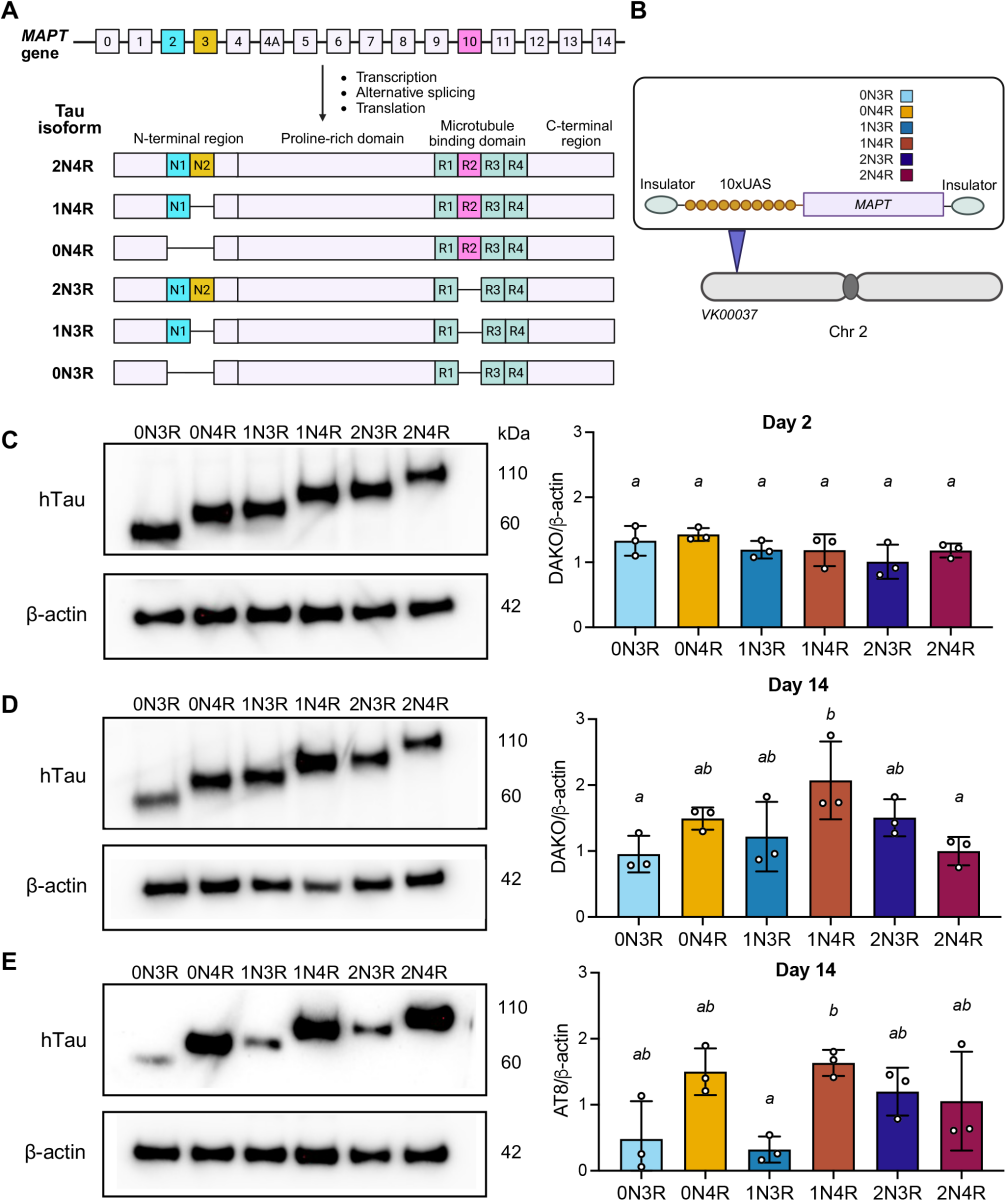
**Generation and validation of transgenic *Drosophila* lines expressing the six human tau (hTau) isoforms*.*** (A) Schematic representation of the human *MAPT* locus and the six hTau isoforms arising from alternative splicing. (B) Overview of the six hTau constructs, each inserted at the VK00037 landing site on chromosome 2. (C-E) Pan-neuronal hTau expression was driven by elav-GAL4, with adult-restricted induction using the TARGET system (tubGal80^ts^*)*; representative western blots are shown on the left and corresponding quantification on the right. (C) Total hTau (DAKO) levels were determined by normalisation to β-actin levels (DAKO/β-actin) at day 2 post-eclosion (*n*=3 independent experiments; mean±s.d.). Letters denote statistical groupings (one-way ANOVA with post-hoc Tukey-corrected multiple comparisons); groups sharing a letter are not significantly different (*P*<0.05). (D) Total hTau at day 14 post-eclosion, showing higher levels of 1N4R accumulation compared to 0N3R and 2N4R. (E) AT8-positive phosphorylated hTau (pTau), showing higher levels for 1N4R than 1N3R. A and B were created in BioRender by Ivanova, Y. (2026). https://BioRender.com/9hzp17n. This figure was sublicensed under CC-BY 4.0 terms.

DNA sequences for each of the six hTau isoforms (Fig. 1A) were placed downstream of ten GAL4 upstream activation sequences (10xUAS). These constructs were flanked by two gypsy insulators and inserted into the same landing site, VK00037*,* on chromosome 2 (Fig. 1B; fly stocks are listed in Table S1). To determine whether hTau isoforms were expressed at comparable levels in adult *Drosophila* central nervous system (CNS), we used the TARGET system, combining the pan-neuronal elav-GAL4 driver with the ubiquitously expressed temperature-sensitive Gal80 repressor (Gal80^ts^) to control transgene induction (McGuire et al., 2003).

Quantification of pan-neuronally expressed hTau by western blotting using the polyclonal pan-Tau antibody DAKO showed that the levels were comparable across isoforms at day 2 post-eclosion (Fig. 1C; Table S2). However, by day 14, isoform-dependent differences in hTau accumulation were evident, with 1N4R showing higher hTau than 0N3R (*P*=0.032) and 2N4R (*P*=0.041) (Fig. 1D). Next, we quantified the levels of phosphorylation at the AT8 epitope site (Ser202/Thr205), which has been widely used as a marker of tau pathology (Goedert et al., 1995). At day 14, AT8-positive hTau (pTau) levels differed across isoforms, with 1N4R showing higher levels than 1N3R (*P*=0.036) (Fig. 1E). Overall, isoform expression levels in our new hTau toolset were comparable in young flies, with isoform-dependent differences in total and AT8-positive hTau evident at later time points.

### hTau isoforms differentially affect fly survival and behaviour

**Fig. 2.**
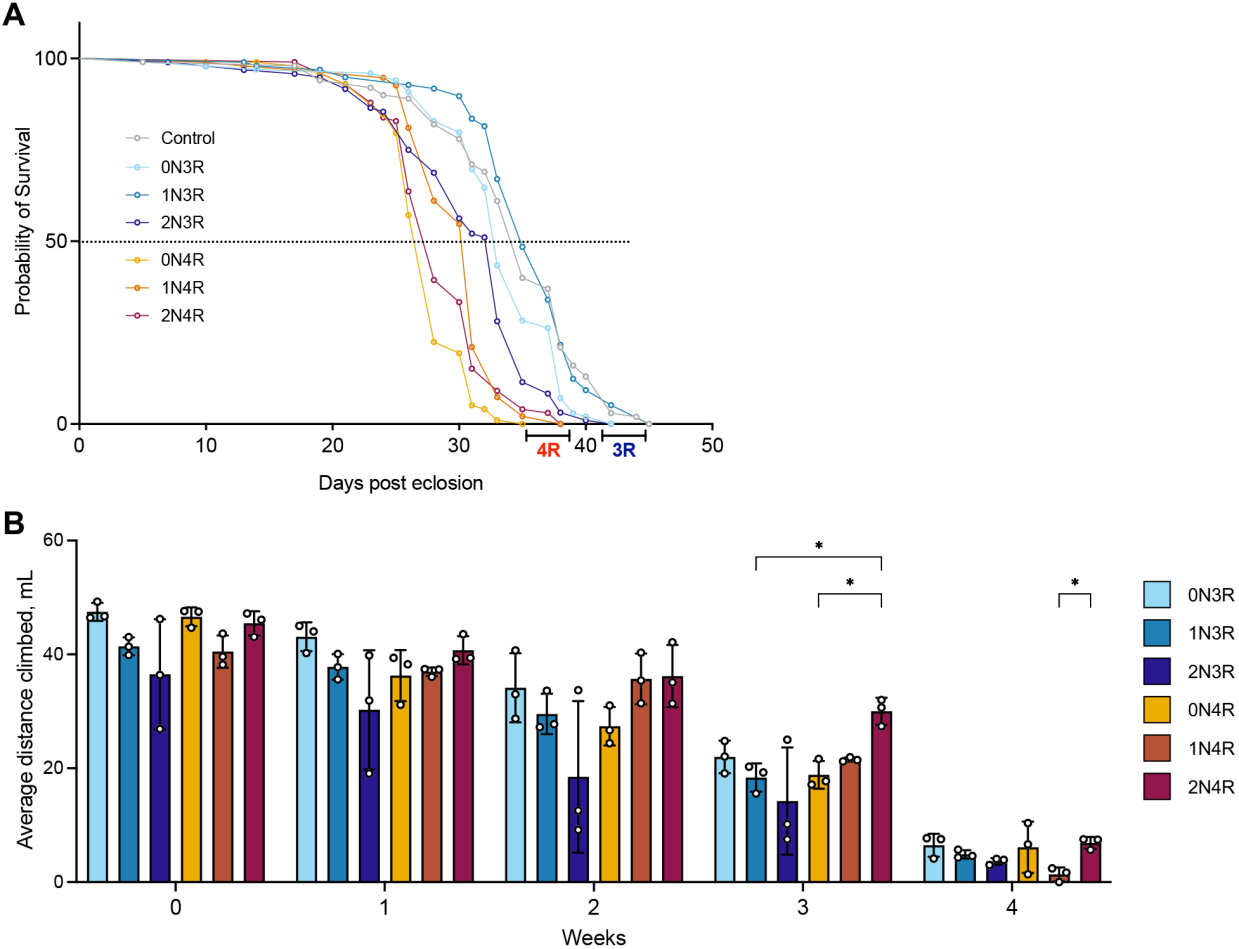
**Behavioural assays demonstrate differential toxicity of the six hTau isoforms.** (A) Lifespan of flies expressing each hTau isoform pan-neuronally (adult-onset induction), shown as Kaplan–Meier survival curves (*n*=100 flies per genotype). Survival differences were analysed using a Cox proportional hazards model with Tukey-corrected post-hoc comparisons. 0N4R was the most toxic isoform in the lifespan assay, followed by 2N4R and 1N4R; 2N3R also reduced lifespan, whereas 0N3R and 1N3R did not. (B) Negative geotaxis (climbing) following adult-onset hTau expression in motor neurons (OK371-GAL4) (*n*=3 cohorts of ten flies per genotype). Climbing distance after 10 s was analysed by repeated-measures two-way ANOVA with Tukey-corrected post-hocs. Climbing performance was broadly comparable across isoforms at early time points. At later time points, flies expressing 2N4R isoform appeared to retain higher climbing performance than 0N4R and 1N3R (week 3), and 1N4R (week 4) (*P<0.05).

The effects of hTau on *Drosophila* lifespan have been well documented (Fernius et al., 2017; Sealey et al., 2017). We next asked whether the pan-neuronal expression of individual hTau isoforms differentially affected lifespan. As measured by the hazard ratio (HR), adult expression of most hTau isoforms significantly reduced *Drosophila* lifespan compared to controls expressing β-galactosidase (LacZ) (likelihood ratio test: χ^2^=307.1, *P*<0.001; Fig. 2A). Of the six isoforms, 0N4R showed the greatest toxicity (HR=8.37, *P*<0.001), followed by 2N4R (HR=5.07, *P*<0.001), 1N4R (HR=4.18, *P*<0.001), and 2N3R (HR=2.28, *P*<0.001). Expression of 0N3R and 1N3R did not reduce lifespan (see Table S3 for all comparisons). Kaplan–Meier median survival estimates and maximum observed survival times for each genotype are provided in Table S4.

Although lifespan analysis provides a measure of overall tau toxicity, it does not capture neuronal dysfunction. Behavioural impairments, including locomotor deficits, are established readouts of tau-induced neuronal dysfunction in *Drosophila* (Ali et al., 2011). To determine whether different hTau isoforms produce different functional impairments, we expressed the isoforms in motor neurons using the OK371-GAL4 driver with developmental expression restricted using the Gal80^ts^ repressor and quantified climbing ability across five weeks (Fig. 2B; Table S5). Climbing performance progressively worsened over time, and, by week 4, flies were almost immobile. The time course of the impairment differed across isoforms [F (11.66, 27.97)=2.243, *P*=0.0395], although isoform-specific differences were only evident at later time points, with 2N4R showing milder toxicity than 0N4R and 1N3R at week 3, and than 1N4R at week 4 (Fig. 2B).

### Differences in the effects of hTau isoforms on degenerative phenotypes

**Fig. 3.**
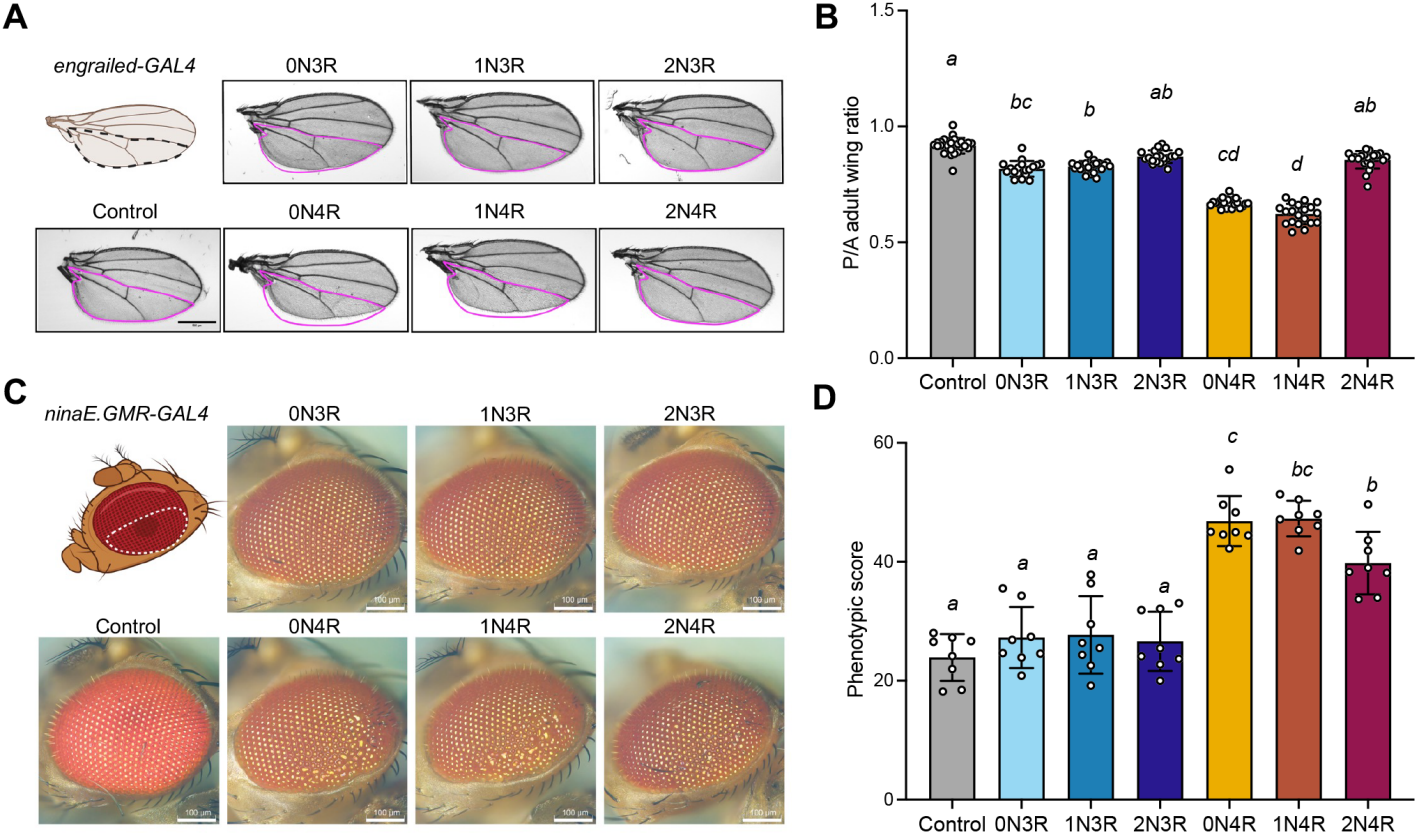
**hTau isoforms cause differential patterns of toxicity depending on tissue.** (A) Toxicity in the wing disc assayed by driving hTau isoform expression in the posterior compartment of the *Drosophila* adult wing using engrailed-GAL4. Flies expressing β-galactosidase were used as controls. Scale bars: 100 μm. (B) Severity of degeneration was quantified with the posterior/anterior (P/A) compartment ratio, with lower values indicating greater impact on the posterior compartment (*n*=27, 18, 23, 20, 23, 20, 21 wing discs; mean±s.d.). Letters denote statistical groupings (Kruskal–Wallis with Dunn's multiple comparisons); groups sharing a letter are not significantly different. All isoforms except 2N3R and 2N4R showed some toxicity compared to β-galactosidase (LacZ) controls. (C) Degeneration in the fly eye following hTau isoform expression driven by ninaE.GMR-GAL4, with β-galactosidase (LacZ) controls. Images were acquired less than 24 h post-eclosion, at 560× total magnification. (D) Degeneration was quantified using the Flynotyper ImageJ plugin and presented as a phenotypic score, with higher values representing greater degeneration (*n*=8, mean±s.d.). Toxicity was observed only with 4R isoforms, with 2N4R resulting in a milder phenotype than 0N4R and 1N4R (one-way ANOVA with Tukey-corrected post-hoc tests). Schematics in A and C were created in BioRender by Ivanova, Y. (2026). https://BioRender.com/pcp7y71. This figure was sublicensed under CC-BY 4.0 terms.

Having established isoform-dependent effects on survival and climbing performance, we then asked how these functional outcomes relate to toxicity phenotypes elsewhere in the fly. As an initial, higher-throughput readout of tissue damage, we leveraged the *Drosophila* wing, which provides a rapid and quantitative *in vivo* platform for assessing tau-induced tissue degeneration (Ramirez-Moreno et al., 2026). Using engrailed-GAL4, each hTau isoform was expressed in the posterior wing compartment, with the anterior compartment serving as an internal control (Fig. 3A). As previously described in Ramirez-Moreno et al. (2026), tau toxicity was quantified as the posterior/anterior (P/A) wing area ratio, which decreases with tissue loss due to degeneration of the targeted posterior compartment tissue. In this assay, hTau toxicity differed across isoforms [Fig. 3B; H(6)=126.4, *P*<0.0001]. Damage was most pronounced with 0N4R and 1N4R, which were equally toxic (Table S6). 0N3R and 1N3R produced more modest, but still detectable, reduction in wing size, while the 2N isoforms (2N3R and 2N4R) showed little impact, the latter consistent with our previous observations (Ramirez-Moreno et al., 2026).

To assess whether the isoform-dependent degeneration patterns observed in the wing generalise to neuronal tissue, we expressed each hTau isoform in the developing visual system using ninaE.GMR-GAL4, which drives expression in photoreceptor cells posterior to the advancing morphogenetic furrow (Freeman, 1996) (Fig. 3C). The adult compound eye is composed of a highly ordered ommatidial lattice, making disruptions to ommatidial organisation a sensitive structural readout of degeneration (Iyer et al., 2016). In the eye, only the 4R isoforms induced ommatidial degeneration [Fig. 3D; F(6, 49)=35.10, *P*<0.0001], whereas 3R isoforms did not produce detectable damage (Table S7). Among the 4R isoforms, 2N4R showed milder toxicity than the others.

### Tau isoforms and selective vulnerability

In Sivanantharajah et al. (2025), we expressed hTau0N3R in small homogeneous populations (hemilineages) of neurons in the adult *Drosophila* CNS and found that some neurons were highly vulnerable to this isoform, leading to severe degeneration, whilst others remained unaffected. To determine if selective vulnerability is driven by the unique properties of individual hTau isoforms or whether all isoforms induce comparable pathology within the same neuron type, we expressed the six hTau isoforms in neurons previously determined to be resilient (19B) and vulnerable (6A) to 0N3R (Meissner et al., 2025). To assess effects of ageing, we compared young adults shortly after eclosion (day 0) with individuals aged to 3 weeks (day 21).

**Fig. 4.**
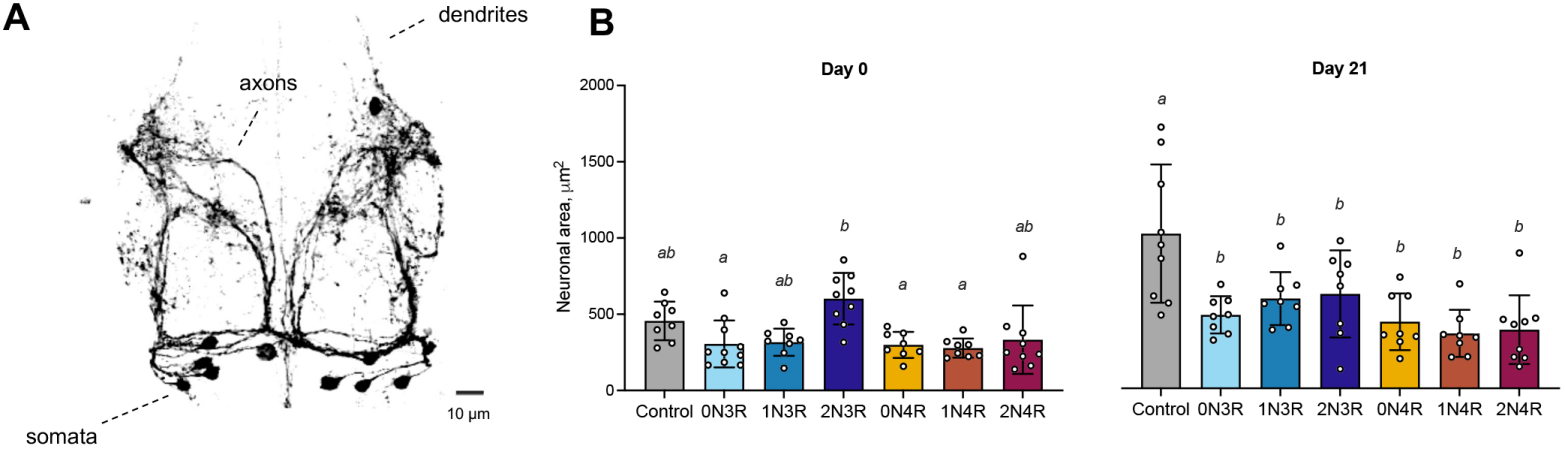
**hTau expression leads to age-associated degeneration even in resilient neurons.** (A) Morphology of neurons previously observed to show resilience to the 0N3R isoform (19B) visualised using mCD8::GFP. (B) Quantification of neuronal area at two time points, day 0 (*n*=8, 10, 8, 9, 8, 8, 9 VNSs; mean±s.d.) and day 21 (*n*=9, 8, 8, 8, 8, 8, 9 VNSs; mean±s.d.) post-eclosion. Individual points represent single VNSs from individual animals. β-galactosidase (LacZ) expression was used as a control. Letters denote statistical groupings; groups sharing a letter are not significantly different (two-way ANOVA with Tukey-corrected post-hoc multiple comparisons, *P*<0.05). hTau expression only resulted in degeneration at day 21, with all isoforms showing similar toxicity.

The 19B hemilineage comprises a small cholinergic population of interneurons originating from the neuroblast (NB) 6-2 (Shepherd et al., 2019). Given the previously demonstrated resilience of 19B to the 0N3R isoform (Sivanantharajah et al., 2025), we assessed whether this effect extended to all six hTau isoforms. To analyse the effects of isoforms on overall 19B neuronal morphology, we labelled cell membranes using membrane-associated green fluorescent protein (mCD8::GFP) and quantified total neuronal area as a morphological readout for degeneration (Fig. 4A). Shortly after eclosion (day 0), morphology of resilient 19B neurons was largely unaffected: none of the hTau isoform-expressing groups showed a significant reduction in neuronal area relative to control (Fig. 4B). At this early time point, we observed small differences in neuronal area between specific isoform pairs (0N3R versus 2N3R, 0N4R versus 2N3R, 1N4R versus 2N3R; Table S8). In contrast, by day 21 post-eclosion, all hTau isoforms produced a comparable reduction in neuronal area relative to the control (*P*<0.003). Thus, 19B neurons show early resilience to hTau-mediated degeneration, but this resilience was not maintained with age. This contrasts with prior work reporting largely normal 19B morphology at 3 weeks following hTau0N3R expression (Sivanantharajah et al., 2025); however, this may be accounted for by the use of a different transgene with differing expression levels.

To explore whether the resilience-to-neurodegeneration transition is accompanied by changes in tau burden (Braak et al., 2006), we quantified total hTau abundance and AT8-positive hTau (pTau) in 19B neurons. Total hTau levels showed no age-dependent increase and were comparable across isoforms and time points (Fig. S1A; Table S2), suggesting that the loss of 19B resilience with age is not readily explained by isoform-dependent differences in hTau accumulation. In contrast, AT8-positive hTau levels were similar across isoforms shortly after eclosion but differed later in life (Table S2). At day 21, two 4R isoforms (0N4R and 1N4R) had higher AT8-positive hTau levels than the corresponding 3R isoforms (0N3R and 1N3R) (Fig. S1B). Together, this indicates that, in the 19B hemilineage, total hTau levels are comparable across isoforms, whereas differences in AT8-positive hTau emerge over time but do not mirror the pattern of degeneration severity.

**Fig. 5.**
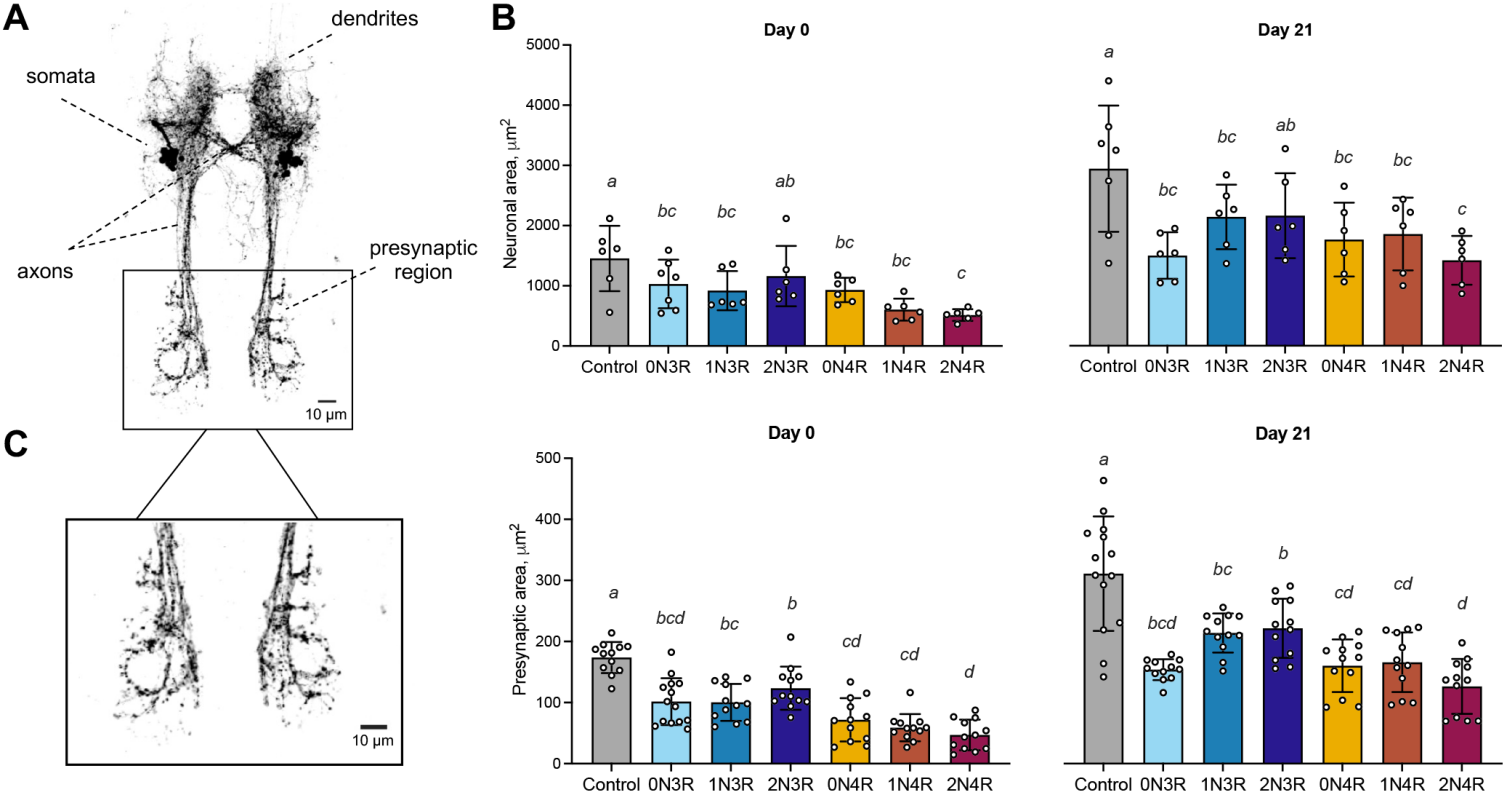
**Isoform-specific patterns of hTau toxicity in vulnerable neurons.** (A) Neuronal morphology of the vulnerable 6A hemilineage visualised using mCD8::GFP. (B) Neuronal area was quantified at day 0 (*n*=6, 7, 6, 6, 6, 6, 6 VNSs; mean±s.d.) and day 21 (*n*=7, 6, 6, 6, 6, 6, 6 VNSs; mean±s.d.) post-eclosion. Individual points represent single VNSs from individual animals. Letters denote statistical groupings; groups sharing a letter are not significantly different (two-way ANOVA with Tukey-corrected post-hoc multiple comparisons; *P*<0.05). Degeneration was observed at both time points for all isoforms except 2N3R. (C) Presynaptic terminal area of 6A neurons was measured as a subcellular readout of toxicity. The presynaptic region used in the analysis is highlighted on the left. Individual points represent quantified presynaptic measurements from the same VNSs analysed in B, with two measurements obtained per VNS. Statistical analysis was performed using a linear mixed-effects model with VNS identity included as a random effect. Although all hTau isoforms resulted in degeneration, 4R isoforms generally exhibited greater toxicity.

We next examined the vulnerable 6A hemilineage to determine whether hTau isoforms differentially affect degeneration severity in a susceptible neuron type. The 6A hemilineage comprises GABAergic interneurons involved in processing sensory feedback during flight-related motor control (Fig. 5A). We measured total neuronal area of 6A neurons as a readout for morphological degeneration (Fig. 5B). In both young and old adults, expression of all hTau isoforms except 2N3R resulted in a significant reduction in neuronal area relative to control (*P*≤0.039; Table S9), indicative of gross neuronal degeneration. Beyond this, isoform-specific differences were limited, with 2N4R showing higher toxicity than 2N3R (*P*=0.033). Together, these data indicate that 6A neurons are broadly susceptible to tau-mediated degeneration across most isoforms, with significant reductions in neuronal area already apparent shortly after eclosion. To assess whether hTau abundance or AT8-positive tau were related to degeneration severity in 6A neurons, we quantified total and AT8-positive hTau levels (Fig. S1C,D; Table S2) and found that both increased over time but remained comparable across isoforms. Therefore, the limited isoform-dependent differences observed in 6A gross morphology were not fully accounted for by total hTau or AT8-positive hTau levels.

Since the 6A neurons are highly polarised with long, regionally specialised structures, whole-cell morphology provides only a coarse readout of degeneration. In our previous work, one of the prominent pathological features in 6A neurons was a reduction in size of distal presynaptic terminals, indicating subcellular, regional sensitivity to degeneration. This is consistent with the broader literature showing that synaptic dysfunction often precedes major structural loss and can serve as a more sensitive indicator of tau-mediated pathology (Scheff et al., 2006). We quantified presynaptic area in 6A neurons (Fig. 5C) and found that all hTau isoforms resulted in reduced presynaptic area relative to control (*P*≤0.002; Table S10). We also observed isoform-specific differences in toxicity: 2N4R produced the greatest presynaptic loss, followed by 0N4R and 1N4R. 0N3R showed an intermediate phenotype, while 1N3R and 2N3R were least toxic. Notably, presynaptic degeneration was detectable even for 2N3R, which showed little impact on gross morphology, underscoring the sensitivity of this presynaptic readout. Overall, our analysis of distinct neuronal subpopulations showed that hTau accumulation and AT8-positive phosphorylation did not consistently predict the extent of degeneration, indicating that neuronal responses to hTau are shaped by other cell-intrinsic factors.

## DISCUSSION

**Fig. 6.**
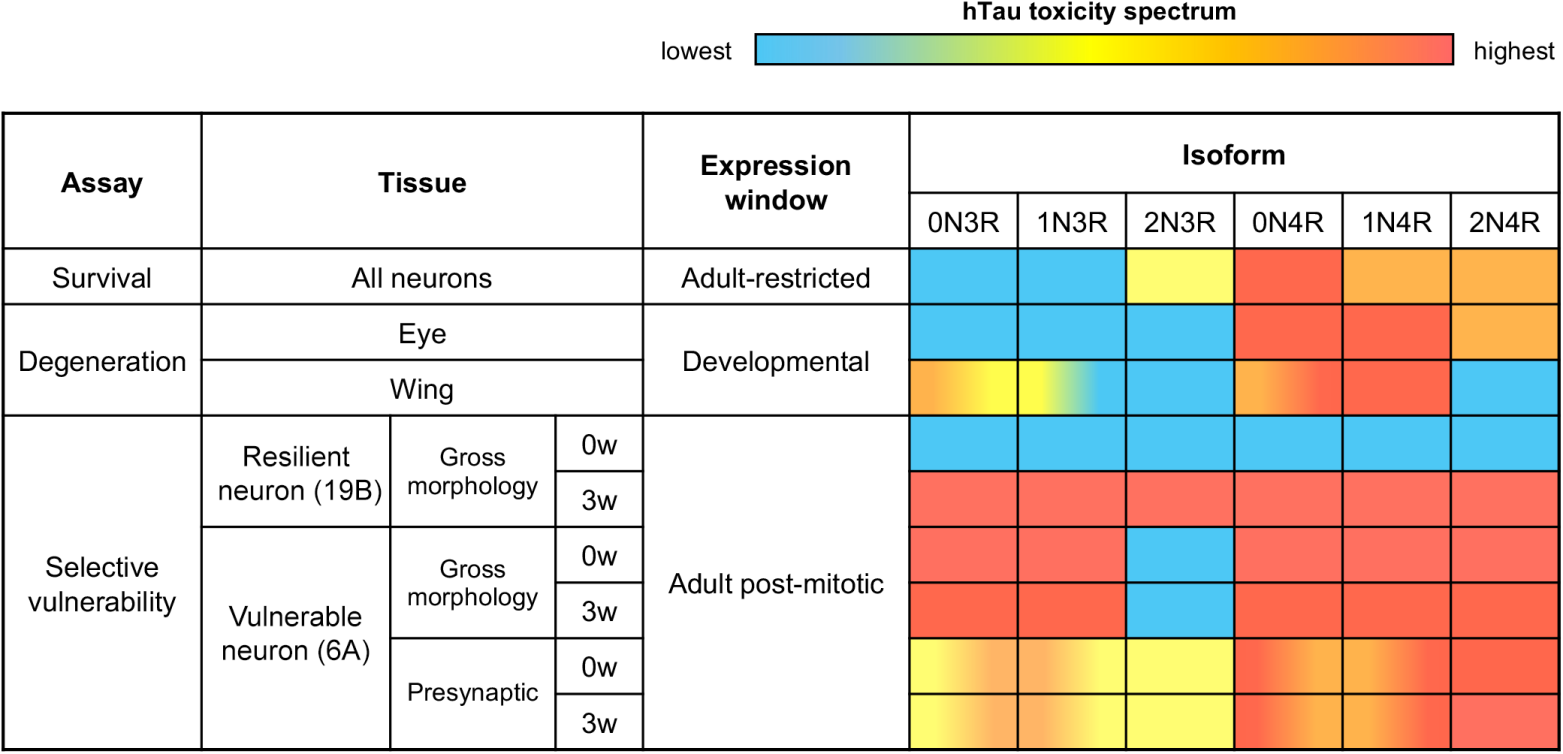
**Summary of results: context-dependent toxicity of the hTau isoforms.** Each cell is coloured according to the post-hoc statistical groupings within that assay (*P*<0.05), providing a categorical summary of relative toxicity rather than a proportional measure of effect magnitude to gauge general isoform-specific differences. Colours were assigned based on the assay metric, including reduced lifespan, increased degeneration, higher phenotypic score, or decreased neuronal area. Red indicates the most toxic group for an assay, and blue denotes no significant difference from the β-galactosidase (LacZ) control. Where a cell contains two colours, the isoform toxicity is not significantly different from either colour group, although these groups differ from each other. No continuous scoring system was used, and no numerical values were assigned to the colours. The expression window indicates whether hTau expression was adult-restricted using the TARGET system, driven by developmental and adult-expressing GAL4 drivers (GMR, en), or confined to post-mitotic neurons using split-GAL4 hemilineage drivers. w, weeks.

Despite tau pathology being a hallmark of many neurodegenerative diseases, the functional differences between tau isoforms and their relative contributions to disease progression remain incompletely understood. This knowledge gap is especially relevant given that distinct tauopathies are characterised by different isoform compositions, with some showing predominant accumulation of 3R tau, 4R tau, or a mixture of both, suggesting that isoform balance may shape clinicopathological heterogeneity. However, most experimental models, including *Drosophila* tauopathy models, typically express a single hTau isoform, and isoform choice varies widely between studies, often without a clear rationale (Cowan et al., 2011). This reductionist approach limits direct, within-systems comparisons and may therefore not capture the full spectrum of isoform-specific toxicity observed in human disease. A comprehensive study of isoform-specific effects is, ultimately, essential for understanding disease heterogeneity and designing targeted interventions. Our study provides a systematic analysis of the toxicity of all six hTau isoforms using a newly developed set of *Drosophila* lines that, by restricting genetic variability, allow clear delineation of isoform-specific differences (summarised in Fig. 6).

We show that, overall, 4R hTau isoforms exhibit markedly higher toxicity than the 3R isoforms. Lifespan analysis revealed a significant reduction with the three 4R isoforms and only 2N3R (Fig. 2A). Interestingly, this contrasts with findings from Fernius et al. (2017), who observed broadly similar effects of all hTau isoforms on lifespan, except for elevated toxicity of 2N3R. Our degeneration assays using the eye and wing readouts also pointed to generally higher toxicity of 4R isoforms. Previous work has shown that expression of hTau during development can be important for triggering degenerative phenotypes (Malmanche et al., 2017). Consistent with this, developmental expression of 4R isoforms driven by GMR-GAL4 resulted in severe degeneration whereas 3R expression did not elicit defects (Fig. 3C). Since the only hTau isoform expressed during human brain development is 0N3R, the extra microtubule-binding repeat in the 4R isoforms may interact with cytoskeletal proteins, drastically altering normal development (Hefti et al., 2018). Nevertheless, the reduction in lifespan we observed when 4R tau expression was restricted to adults using the TARGET system indicates that developmental expression is not essential for tau-mediated toxicity and can occur in adult flies as a function of age.

Adult-restricted expression using the TARGET system requires transfer from 18°C to 29°C, so TARGET-based adult phenotypes should be interpreted in the context of a shared temperature-shift paradigm that may influence stress response and proteostasis pathways (Bettencourt et al., 2008; Donovan and Marr, 2016). Because this shift was applied across all genotypes, including controls, it is unlikely to account for isoform-specific differences by itself. Importantly, previous TARGET-based work has shown that adult induction of 4R hTau expression does not necessarily produce detectable degenerative phenotypes (Malmanche et al., 2017), suggesting that the emergence of toxicity depends on robust hTau expression together with isoform-specific properties, rather than heat-shock priming alone.

The higher toxicity of 4R isoforms aligns well with the isoform imbalance observed in human tauopathies. Primary tauopathies, such as PSP, CBD, and many others are characterised by pathological accumulation of 4R tau, whereas 3R-dominant pathology is rare (Murray et al., 2014; Mailliot et al., 1998). While this supports the relevance of isoform-specific toxicity, isolated isoform expression in *Drosophila* does not fully capture human tauopathies, where multiple tau isoforms often coexist and undergo disease stage-dependent biochemical changes. One possible explanation for the 3R-4R differences observed here is the presence of an additional hexapeptide aggregation motif, VQIINK, in the R2 repeat of 4R tau (von Bergen et al., 2001). VQIINK can form highly stable steric zippers leading to strong self-aggregation capacity (Seidler et al., 2018). Although VQIVYK is essential for filament formation, VQIINK deletion has been linked to reduced tau assembly, raising the possibility that VQIINK may contribute to enhanced 4R toxicity (Zhang et al., 2019). This remains a mechanistic hypothesis, however, and was not directly tested in the present study.

Although 4R isoforms showed greater toxicity overall, the pattern across our phenotypic measures was more nuanced than a simple 3R-4R dichotomy, with meaningful isoform-specific differences emerging across assays. Analysis of hTau-induced functional deficits using negative geotaxis revealed some isoform-specific effects, although these differences only emerged at later weeks (Fig. 2B). Notably, the 2N4R isoform had relatively preserved climbing performance at later time points despite showing reduced survival, suggesting that locomotor dysfunction and organismal viability do not necessarily deteriorate in parallel. We also did not detect increased toxicity of 0N3R relative to 0N4R, in contrast to findings reported by Sealey et al. (2017), indicating that isoform-dependent effects can vary by phenotype and experimental context.

Importantly, our degeneration assays in the wing and the eye showed considerable variation in toxicity within both the 3R and 4R groups. In both tissues, 2N isoforms containing both N-terminal inserts (2N) produced no obvious degeneration (2N3R in the eye, and 2N3R/2N4R in the wing). This suggests important modulatory contributions of the regions outside the microtubule-binding domains. The functional profile of the N-terminal region appears highly complex, with documented involvement in modulating microtubule stabilisation (e.g. bundle formation/spacing), regulation of axonal transport, and interactions with plasma membrane (Derisbourg et al., 2015), (Brandt et al., 1995). Structurally, the N-terminal region is intrinsically disordered, which may facilitate diverse intermolecular interactions, influencing the ability of tau to engage in complex regulatory networks or dynamic assemblies. Tau structural models, such as the ‘paperclip’ conformation, propose that the N- and the C-termini fold back onto the repeat domain, with the number of the N-terminal inserts modulating the protein conformation (Jeganathan et al., 2006; Verelst et al., 2020). Together, these observations support the idea that the N-terminal composition can tune the conformational ensemble of tau and its interaction landscape, shaping whether toxicity leads to overt degeneration or is buffered despite belonging to the same 3R or 4R group.

Taken together, our findings also suggest that isoform toxicity is shaped by tissue-specific factors. In the wing, damage was most pronounced with the 0N4R and 1N4R isoforms, whereas 0N3R and 1N3R were less toxic but still produced a detectable posterior-compartment phenotype (Fig. 3B). In contrast, in the neuronal context in the eye, only 4R isoforms induced ommatidial degeneration, while 3R isoforms produced no detectable damage (Fig. 3D). Overall, these tissue-specific differences in toxicity indicate that local cellular environments may influence the effects of tau. One possible explanation is that tissues differ in the relative abundance and activity of tau modifiers and interactors (e.g. phosphorylation machinery, cytoskeletal pathways, quality-control pathways etc.). These tissue-specific factors are likely to shape selective vulnerability: the same tau isoform may be tolerated in some cell populations yet drive degeneration in others. Isoform-dependent toxicity would then emerge from the interaction between tau structure and cell-type-specific molecular landscapes.

To study neurodegeneration in the context of selective vulnerability, we restricted hTau isoform expression to small, highly homogeneous neuronal populations in the fly CNS that were previously identified as vulnerable or resilient to the 0N3R hTau isoform (Sivanantharajah et al., 2025). This approach offers several advantages. First, these hemilineages are anatomically well characterised, enabling reliable identification of distinct subcellular compartments. Another major advantage of our system is its resolution. Given that degeneration may be first detected in particularly vulnerable neuronal regions (e.g. synaptic terminals), subcellular analysis of neuronal morphology may reveal differences that are not captured by gross area measurements. The strengths of this system were further enhanced by our hTau isoform panel, in which each transgene was inserted at a defined landing site to reduce expression-related confounds and improve comparability between the lines.

The resilient 19B and vulnerable 6A hemilineages showed fundamentally different temporal responses to hTau expression. In 19B neurons, isoform-specific toxicity was minimal shortly after eclosion: no isoform produced clear degenerative effects at this early stage, and only subtle differences were detected between a small number of isoform pairs (Fig. 4). By 3 weeks, however, this early resilience was lost and all isoforms converged on a similar toxic outcome, with little evidence for sustained isoform selectivity. This suggests that intrinsic factors may initially restrain toxicity in 19B neurons, but this protection can diminish with age as additional cellular stressors accumulate. This transition from resistance to vulnerability may parallel the hierarchical progression described by Braak staging (Braak et al., 2006), where tau pathology emerges in selective regions before spreading to broader cortical territories. Age-dependent breakdown of intrinsic constraints could therefore help explain why certain neuronal populations become permissive to pathology only at later stages of disease.

In contrast, 6A neurons showed a rapid and severe response to hTau expression, with degeneration evident from the earliest time point and persisting at 3 weeks (Fig. 5). Isoform-dependent differences were limited, as all isoforms were toxic except for 2N3R, and 2N4R showed greater toxicity than 2N3R. Importantly, this pattern was not fully accounted for by detectable total hTau signal or AT8-positive tau levels alone. Rather than reflecting detectable tau levels or phosphorylation at a single disease-relevant epitope, it is possible that toxicity depends on how tau behaves within a given neuronal context: its subcellular localisation, conformational state, broader post-translational modification profile at other disease-relevant phospho-sites, and interactions with cell-type-specific pathways.

This context-dependent view of tau toxicity highlights the importance of subcellular analysis, as differences in tau behaviour may become more apparent in particularly vulnerable neuronal regions. In the presynaptic compartment, we observed clear isoform-dependent stratification in toxicity. 2N4R was the most toxic isoform, followed by modest toxicity of 0N4R and 1N4R, intermediate toxicity of 0N3R, and mild toxicity of 1N3R and 2N3R (Fig. 5C). These findings directly further our previous work on selective vulnerability and show that both neuronal identity and isoform specificity determine the severity of pathological response. Furthermore, the sensitivity of 6A presynaptic structures to tau-mediated degeneration underscores the value of subcellular resolution in investigating early neuronal pathology.

Some limitations should be considered when interpreting these findings. First, total hTau measurements relied on the DAKO/Agilent polyclonal anti-tau antibody and therefore represent detectable protein signal with this reagent rather than definitive measures of absolute isoform abundance. Isoform-dependent differences in epitope availability or antibody-binding efficiency cannot be fully excluded. Second, AT8, which is a widely used marker of disease-associated tau, was used to assess phosphorylation at Ser202/Thr205. However, AT8 represents one disease-relevant phospho-epitope, and it is possible that phosphorylation at other sites or other post-translational modifications not explored here may also contribute to isoform-specific toxicity. Third, the wing and eye assays provide phenotypic readouts of isoform-dependent toxicity; however, they were not accompanied by tissue-specific biochemical analysis here. While tissue-specific modification of tau may contribute to these phenotypes, the consistent emergence of stronger 4R-associated toxicity across multiple assays and tissue types suggests that these factors are unlikely to fully account for the overall 3R/4R pattern. Finally, all assays were performed in males, with the exception of 19B and 6A ventral nervous system (VNS) immunohistochemistry experiments, which were performed in females to maintain consistency with previously published studies (Sivanantharajah et al., 2025). Potential sex-dependent modulation of isoform-specific toxicity was not evaluated in this study.

Overall, the hTau isoform line set generated here offers a tractable *in vivo* platform for dissecting isoform-specific tau toxicity. Using this platform, we identify a context-dependent split in isoform toxicity, with 4R isoforms generally conferring higher toxicity than 3R isoforms, yet with substantial heterogeneity across measures, tissues, and neuronal populations. Future work could now use this platform to investigate the 4R-specific features (e.g. R2 including the VQIINK motif) and to systematically define isoform-specific post-translational modification landscapes that may modulate subcellular localisation, aggregation rate, and neurotoxicity. More broadly, this set of six hTau isoform *Drosophila* lines provides a foundation for scalable candidate and unbiased modifier approaches to map the cellular pathways that buffer or exacerbate isoform-specific toxicity.

## MATERIALS AND METHODS

### *Drosophila* culture

Flies were raised on a standard diet of cornmeal with a 12 h light: 12 h dark cycle. For experiments using the TARGET system (McGuire et al., 2004) to restrict gene expression to adult neurons (e.g. western blotting, survival, negative geotaxis), crosses were reared at 18°C and flies of relevant genotypes moved to 29°C upon eclosion. Crosses using the ninaE.GMR-GAL4 driver to examine rough eye phenotypes were reared at 25°C. Details of all *Drosophila* stocks used in this study are provided in Table S1.

### Transgenic hTau isoform flies

To create new hTau-expressing lines, DNA sequences for each of the six hTau isoforms were placed downstream of ten GAL4 upstream activation sequences (10xUAS). These constructs were insulated with two *gypsy* insulators and inserted into chromosome 2 at the same landing site, VK00037 (Fig. 1B). Cloning was completed by GenScript (NJ, USA) and the injection and creation of stable transgenic lines by GenomeProlab (Montreal, Canada). The newly generated UAS-hTau isoform *Drosophila* lines are available from the corresponding author upon request.

### Western blotting to quantify transgene expression

To compare hTau expression across the new transgenic lines, expression of each isoform was driven using the pan-neuronal driver, elav-GAL4 and tubGal80^ts^ to restrict expression to adult flies. Crosses were set up at 18°C, and, upon eclosion, male *Drosophila* were collected and aged at 29°C for 2 days. Flies were decapitated and stored at −80°C until use. Frozen heads were placed in ice-cold homogenisation buffer (HB): 10 mM Tris, 150 mM NaCl, 5 mM EDTA, 20% v/v glycerol, protease and phosphatase inhibitors (1:100). Fly heads were manually homogenised for 3 min in HB (5 μl/head). Lysates were centrifuged at 9600 ***g*** at 4°C for 5 min. The supernatant was removed, mixed with NuPAGE LDS sample buffer (NP0007, Thermo Fisher Scientific) and DTT and boiled at 95°C for 5 min. Western blotting was performed using standard protocols (Mahmood and Yang, 2012).

Primary antibodies used in this study were as follows: rabbit anti-hTau (Agilent-DAKO, A002401-2) at 1:15,000 dilution, mouse anti-phospho-Tau (Ser202, Thr205) antibody (AT8, Fisher, 10599853) at 1:1000 dilution, and mouse anti-β-actin antibody (Abcam, ab8224) at 1:3000 dilution. Secondary antibodies used were anti-rabbit HRP-conjugated (Invitrogen, 31460) and anti-mouse HRP-conjugated (Invitrogen, 31430). Antibody-antigen complexes were detected using Chemiluminescent Peroxidase Substrate-3 (Sigma-Aldrich, CPS350-1KT). Blots were imaged on a Bio-Rad ChemiDoc system. Protein expression levels were quantified by densitometric analysis of bands using ImageJ [i.e. FIJI (http://rsbweb.nih.gov/ij/)].

### Survival assay

To determine the effect of each isoform on fly lifespan, the TARGET system was used with pan-neuronal elav-GAL4 to restrict expression of transgenes to adult flies (McGuire et al., 2003). Elav-GAL4, tubGal80^ts^ flies were crossed to each of the six UAS-hTau isoform lines and UAS-lacZ (Bloomington *Drosophila* Stock Center #8529; Table S1). LacZ was used as a non-tau UAS transgene control to distinguish hTau-associated phenotypes from effects related to GAL4/UAS-driven transgene expression alone (Martin-Pena et al., 2018; Xu et al., 2015). Crosses were maintained at 18°C to suppress any transgene expression. Upon eclosion, male progeny were collected and transferred to 29°C (ten flies per vial). Flies were transferred to new vials every 2-3 days, and the number of surviving flies was recorded. Kaplan–Meier curves were generated using GraphPad Prism (version 10.6; GraphPad Software, 2025). Survival was analysed in R using a Cox proportional hazards model: Surv(Day, Status)∼Genotype+frailty(Vial).

### Negative geotaxis assay

To examine the effects of isoform expression on climbing behaviour, transgenic UAS-hTau *Drosophila* lines were crossed to the motor neuron-specific OK371-GAL4 driver. Expression of hTau was restricted to adults using the TARGET system (McGuire et al., 2003). Male progeny were collected and transferred to 29°C (ten flies per vial). On the day of the experiment, flies were flipped into cylinders and allowed to acclimatise to the room temperature for 1 h. The vials were gently tapped, and the distance climbed by each fly after 10 s was recorded. This procedure was repeated three times with 30 s breaks in between the measurements.

### *Drosophila* wing assay

To examine hTau isoform toxicity in a tissue-level degeneration assay, we drove expression of isoforms using the engrailed-GAL4 driver (Tabata et al., 1995), which drives transgene expression in the posterior compartment of the wing. Flies were reared at 25°C, and male progeny were collected up to 48 h after eclosion and frozen. Wings were removed and imaged using a Leica Stereoscopic microscope (LAS Software). The ratio of posterior to anterior wing compartments was calculated as described in Ramirez-Moreno et al. (2026) with Fiji.

### Eye phenotype analysis

To determine if different hTau isoforms could elicit a rough eye phenotype, we used the ninaE.GMR-GAL4 driver. Male progeny were collected less than 24 h post-eclosion and frozen. *Drosophila* eyes were imaged using a DSX-1000 microscope (Olympus) with a 40× objective and the final magnification of 560×. Flynotyper plugin in ImageJ (https://flynotyper.sourceforge.net/imageJ.html) was used to calculate phenotypic scores, with higher values indicating greater degeneration.

### VNS tissue immunohistochemistry and confocal microscopy

Flies for these experiments were set at 18°C^,^ and the female progeny were transferred to 29°C after eclosion. VNSs were dissected and processed as described in Shepherd et al. (2019). Primary antibodies used were as follows: 1:1000 chicken anti-GFP (Invitrogen-Molecular Probes, A10262), 1:15,000 rabbit anti-hTau (Agilent-DAKO, A002401-2), 1:125 mouse anti-lacZ (J1E7, DSHB); 1:1000 mouse anti-phospho-Tau (Ser202, Thr205) antibody (AT8, Fisher, 10599853). Relevant secondary antibodies were used at 1:500. Confocal microscopy images were taken using a Zeiss LSM 710 confocal microscope. Images used for signal quantification were obtained at 40× oil magnification using identical image acquisition parameters. Image acquisition and quantification were performed while aware of genotype.

### Analysis of neuronal morphology

Neuronal morphology was visualised using the mCD8-GFP signal. Neuronal area was measured using the maximum intensity projections of the neuronal images. For the subcellular morphological analysis, two measurements per VNS were made, with six VNSs analysed per isoform. Statistical analysis was performed in R using a linear mixed effects model (package ‘lme4’, v.1.1.35.5). Isoform, time, and their interaction were modelled as fixed effects, and the VNS identity was included as a random effect: lmer(Area∼Isoform*Time+(1|Subject)). Total hTau and AT8-positive hTau levels were measured as corrected total cell fluorescence (CTCF), where CTCF=Integrated Density–(Area of selected cell×Mean fluorescence of background readings) (McCloy et al., 2014).

### Statistical analysis

Unless stated otherwise, all statistical analyses were performed in GraphPad Prism (version 10.6; GraphPad Software, 2025). A Kruskal–Wallis test with post-hoc Dunn's tests was used to evaluate the differences in P/A wing ratio (Table S6). Repeated measures two-way ANOVA with Tukey-corrected post-hoc tests was used to analyse the effects of isoforms on climbing ability (Table S5). Due to the longitudinal structure of the climbing dataset, the Geisser-Greenhouse correction was applied to account for potential violations of sphericity. One-way ANOVAs with Tukey-corrected post-hoc tests were used to assess the differences in the eye phenotypic score between the isoforms (Table S7). Two-way ANOVAs with Tukey-corrected post-hoc tests were used to analyse the effects of isoform expression, time, and their interaction on neuronal area, total hTau, and AT8-positive hTau levels (Fig. S1).

## Supplementary Material



10.1242/biolopen.062644_sup1 Supplementary information
