# Supplementary material for: Context-dependent toxicity of human tau isoforms in a Drosophila tauopathy model
Source: Biol Open. 2026 Jul 28;15(7):bio062644. doi: 10.1242/bio.062644 (PMC13474566; doi:10.1242/bio.062644)
Supplement: Supplementary file 1 [file biolopen-15-062644-s1.pdf]

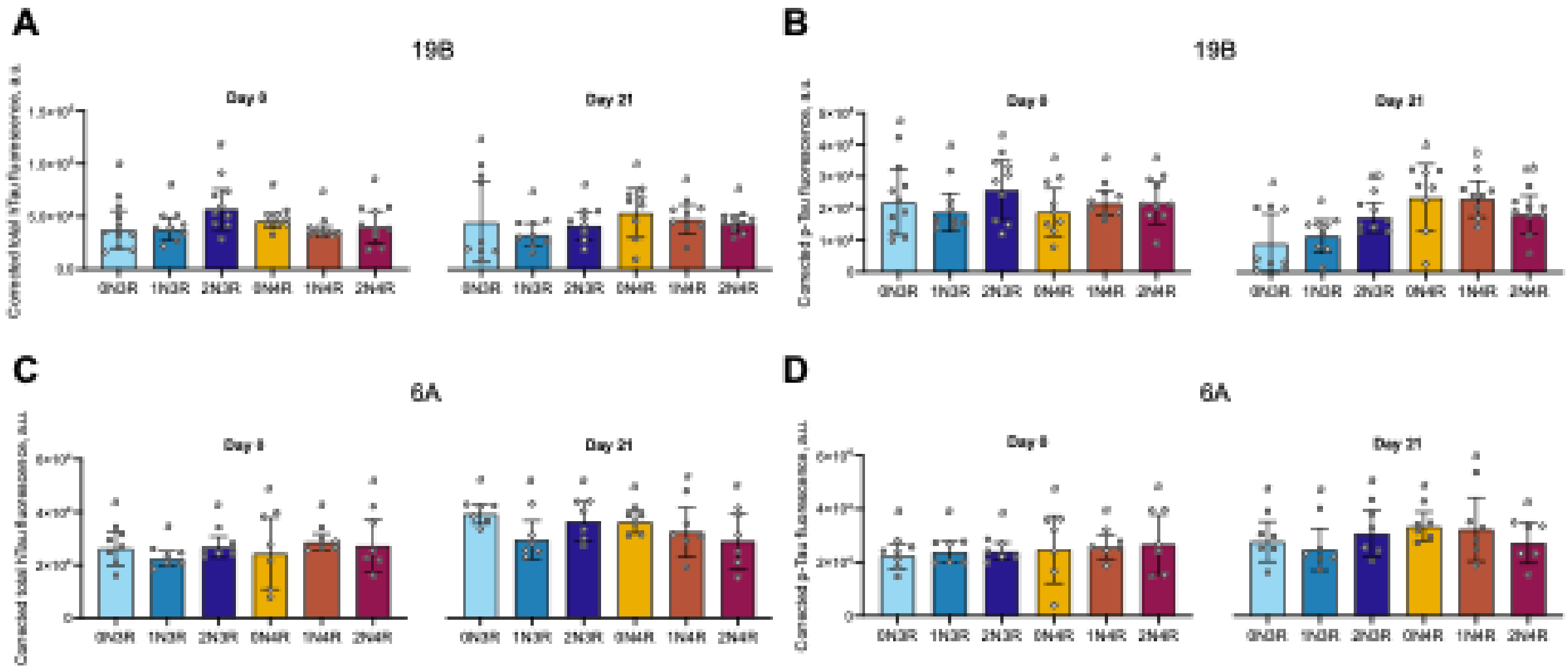

Fig. S1. Quantification of total hTau and AT8-positive hTau (p-Tau) levels in resilient (19B) and vulnerable (6A) neurons. The levels were quantified as corrected total cell fluorescence at day 0 and day 21 post-eclosion. **A, B** For 19B neurons, n = 8, 10, 8, 9, 8, 8 and 9 VNSs at day 0, and n = 9, 8, 8, 8, 8, 8 and 9 VNSs at day 21; mean ± SD. **C, D** For 6A neurons, n = 6, 7, 6, 6, 6, 6 and 6 VNSs at day 0, and n = 7, 6, 6, 6, 6, 6 and 6 VNSs at day 21; mean ± SD. Statistical significance was assessed using two-way ANOVA with post-hoc Tukey-corrected multiple comparisons; groups sharing a letter are not significantly different ($p < 0.05$). **A** Total hTau levels in resilient 19B neurons were broadly comparable across isoforms and did not increase with age. **B** AT8-positive hTau levels in the same neurons were similar across isoforms at day 0, but by day 21 isoform-specific differences emerged, with 0N4R and 1N4R showing higher levels than 0N3R and 1N3R. **C, D** In vulnerable 6A neurons, total hTau and AT8-positive levels both increased over time but remained comparable across isoforms.

Biology Open • Supplementary information

Table S1. *Drosophila* stocks used in this study.

**Fly line**	**Genotype**	**Source**	**Use**
hTau0N3R.VK37	+; *UAS-hTau0N3R.VK00037/CyO;+*	This paper	All assays
hTau0N4R.VK37	+;*UAS-hTau0N4R.VK00037/CyO;+*	This paper	All assays
hTau1N3R.VK37	+; *UAS-hTau1N3R.VK00037/CyO*	This paper	All assays
hTau1N4R.VK37	*; UAS-hTau1N4R.VK00037/CyO*	This paper	All assays
hTau2N3R.VK37	*; UAS-hTau2N3R.VK00037/CyO*	This paper	All assays
hTau2N4R.VK37	*; UAS-hTau2N4R.VK00037/CyO*	This paper	All assays
lacZ	*; UAS-lacZ/CyO; MKRS/TM6B*	BDSC no. 8529	Control
elav-GAL4, $tubGal80^{ts}$	*elav-GAL4; + ;*$tubGal80^{ts}$	BDSC no. 458	Survival, western blotting
OK371, $tubGal80^{ts}$	*; OK371;* $tubGal80^{ts}$	BDSC no. 26160	Negative geotaxis
GMR-GAL4	*; GMR-Gal4/CyO*	BDSC no. 9146	Eye phenotype analysis
engrailed-GAL4	*; engrailed2.4-GAL4e16E*	BDSC no. 30564	Wing assay
6A-GAL4.GFP	*; 6A-GAL4, mCD8::GFP/CyO; 6A-GAL4/MKRS*	Modified from 6A-GAL4 (SS26967, FlyLight, Janelia)	Selective vulnerability
19B-GAL4.GFP	*; 19B-GAL4, mCD8::GFP/CyO; 19B-GAL4/MKRS*	Modified from 19B-GAL4 (SS20748, FlyLight, Janelia)	Selective vulnerability

Biology Open • Supplementary information

Table S2. Omnibus test statistics and effect size estimates for hTau and AT8-positive Tau measurements

Dataset	Measurement	Effect tested	Test statistic	Effect size	95% CI	p-value
Fig. 1C	Total hTau, day 2	Isoform	$F(5, 12) = 1.683$	$\eta^2 = 0.4122$	[0.0000, 0.6402]	0.2131
Fig. 1D	Total hTau, day 14	Isoform	$F(5, 12) = 3.612$	$\eta^2 = 0.6008$	[0.0000, 0.7402]	0.0317
Fig. 1E	AT8-positive hTau	Isoform	$F(5, 12) = 4.178$	$\eta^2 = 0.6351$	[0.0303, 0.7971]	0.0197
Fig. S1A	Total hTau	Isoform x Time interaction	$F(5, 89) = 1.412$	$\eta p^2 = 0.0735$	[0.0000, 0.1465]	0.2276
		Isoform	$F(5, 89) = 1.598$	$\eta p^2 = 0.0824$	[0.0000, 0.1596]	0.1689
		Time	$F(1, 89) = 0.1433$	$\eta p^2 = 0.0016$	[0.0000, 0.0538]	0.7059
Fig. S1B	AT8-positive hTau	Isoform x Time interaction	$F(5, 89) = 3.169$	$\eta p^2 = 0.1511$	[0.0099, 0.2604]	0.0112
		Isoform	$F(5, 89) = 2.936$	$\eta p^2 = 0.1416$	[0.0048, 0.2487]	0.0168
		Time	$F(1, 89) = 8.660$	$\eta p^2 = 0.0887$	[0.0096, 0.2153]	0.0041
Fig. S1C	Total hTau	Isoform x Time interaction	$F(5, 61) = 1.115$	$\eta p^2 = 0.0837$	[0.0000, 0.1815]	0.3618
		Isoform	$F(5, 61) = 1.345$	$\eta p^2 = 0.0993$	[0.0000, 0.2059]	0.2579
		Time	$F(1, 61) = 19.64$	$\eta p^2 = 0.2436$	[0.0801, 0.4112]	< 0.0001
Fig. S1D	AT8-positive hTau	Isoform x Time interaction	$F(5, 61) = 0.4934$	$\eta p^2 = 0.0389$	[0.0000, 0.0938]	0.7799
		Isoform	$F(5, 61) = 0.7552$	$\eta p^2 = 0.0583$	[0.0000, 0.1363]	0.5855
		Time	$F(1, 61) = 6.720$	$\eta p^2 = 0.0992$	[0.0049, 0.2571]	0.0119

Biology Open • Supplementary information

Table S3. Tukey-corrected post-hoc multiple comparisons for survival assay

	0N3R	1N3R	2N3R	0N4R	1N4R	2N4R
Control	0.1174	0.9977	<0.0001	<0.0001	<0.0001	<0.0001
0N3R		0.0248	0.0424	<0.0001	<0.0001	<0.0001
1N3R			<0.0001	<0.0001	<0.0001	<0.0001
2N3R				<0.0001	0.0008	<0.0001
0N4R					<0.0001	0.0099
1N4R						0.8395

Table S4. Kaplan-Meier median survival estimates and maximum observed survival time by genotype

Genotype	Median survival time, days (95% confidence intervals)	Maximum observed survival time (days)
Control (lacZ)	35 (not estimable)	45
0N3R	33 (33-35)	42
1N3R	35 (35-37)	45
2N3R	33 (30-33)	42
0N4R	28 (26-28)	35
1N4R	31 (30-31)	38
2N4R	28 (not estimable)	38

Table S5. Tukey-corrected post-hoc multiple comparisons for negative geotaxis assay (all timepoints shown)

Week 0

	1N3R	2N3R	0N4R	1N4R	2N4R
0N3R	0.0506	0.5439	0.9786	0.1407	0.7696
1N3R		0.9263	0.0907	0.9926	0.2809
2N3R			0.5953	0.9691	0.6733
0N4R				0.1927	0.9652
1N4R					0.3331

Week 1

	1N3R	2N3R	0N4R	1N4R	2N4R
0N3R	0.2516	0.4995	0.3880	0.1592	0.6859
1N3R		0.8091	0.9901	0.9795	0.6746
2N3R			0.9183	0.8515	0.6287
0N4R				0.9995	0.6859
1N4R					0.3670

Biology Open • Supplementary information

Biology Open (2026): doi:10.1242/bio.062644: Supplementary information

Week 2

	1N3R	2N3R	0N4R	1N4R	2N4R
0N3R	0.8454	0.5498	0.6097	0.9985	0.9964
1N3R		0.7423	0.9613	0.5190	0.5705
2N3R			0.8458	0.4705	0.4591
0N4R				0.2907	0.3596
1N4R					>0.9999

Week 3

	1N3R	2N3R	0N4R	1N4R	2N4R
0N3R	0.6104	0.7510	0.7061	0.9994	0.1087
1N3R		0.9597	0.9998	0.4699	0.0239
2N3R			0.9406	0.7621	0.3170
0N4R				0.5588	0.0268
1N4R					0.0820

Week 4

	1N3R	2N3R	0N4R	1N4R	2N4R
0N3R	0.7825	0.4203	>0.9999	0.1298	0.9991
1N3R		0.3728	0.9928	0.0956	0.2660
2N3R			0.9061	0.2593	0.0800
0N4R				0.5966	0.9994
1N4R					0.0251

Table S6. Dunn’s multiple comparisons for the P/A adult wing ratio

	0N3R	1N3R	2N3R	0N4R	1N4R	2N4R
Control	<0.0001	<0.0001	0.5078	<0.0001	<0.0001	0.0883
0N3R		>0.9999	0.1632	0.1237	0.0054	0.6301
1N3R			0.3888	0.0131	0.0003	>0.9999
2N3R				<0.0001	<0.0001	>0.9999
0N4R					>0.9999	<0.0001
1N4R						<0.0001

Table S7. Tukey-corrected post-hoc multiple comparisons for eye degeneration

	0N3R	1N3R	2N3R	0N4R	1N4R	2N4R
Control	0.8070	0.7006	0.9174	<0.0001	<0.0001	<0.0001
0N3R		>0.9999	>0.9999	<0.0001	<0.0001	<0.0001
1N3R			0.9993	<0.0001	<0.0001	0.0002
2N3R				<0.0001	<0.0001	<0.0001
0N4R					>0.9999	0.0728
1N4R						0.0469

Biology Open • Supplementary information

Table S8. Tukey-corrected post-hoc multiple comparisons for the neuronal area of the 19B resilient neurons

Week 0

	0N3R	1N3R	2N3R	0N4R	1N4R	2N4R
Control	0.7198	0.8250	0.7712	0.7320	0.6005	0.8824
0N3R		>0.9999	0.0359	>0.9999	>0.9999	>0.9999
1N3R			0.0761	>0.9999	0.9998	>0.9999
2N3R				0.0485	0.0268	0.0936
0N4R					>0.9999	0.9999
1N4R						0.9980

Week 21

	0N3R	1N3R	2N3R	0N4R	1N4R	2N4R
Control	<0.0001	0.0009	0.0028	<0.0001	<0.0001	<0.0001
0N3R		0.9455	0.8365	0.9994	0.9022	0.9600
1N3R			>0.9999	0.7617	0.3013	0.4034
2N3R				0.5725	0.1687	0.2377
0N4R					0.9900	0.9986
1N4R						>0.9999

Table S9. Tukey-corrected post-hoc multiple comparisons for the neuronal area of the 6A vulnerable neurons

	0N3R	1N3R	2N3R	0N4R	1N4R	2N4R
Control	0.0006	0.0390	0.1702	0.0029	0.0004	<0.0001
0N3R		0.8754	0.5155	0.9997	>0.9999	0.8036
1N3R			0.9965	0.9796	0.8107	0.1453
2N3R				0.7753	0.4341	0.0328
0N4R					0.9982	0.5884
1N4R						0.8890

Table S10. Tukey-corrected post-hoc multiple comparisons for presynaptic area of 6A vulnerable neurons

	0N3R	1N3R	2N3R	0N4R	1N4R	2N4R
Control	<0.0001	0.0001	0.0019	<0.0001	<0.0001	<0.0001
0N3R		0.6000	0.1306	0.9930	0.9714	0.1323
1N3R			0.9728	0.2259	0.1473	0.0010
2N3R				0.0268	0.0146	<0.0001
0N4R					1.0000	0.4894
1N4R						0.6290
