# Supplementary material for: Context-dependent toxicity of human tau isoforms in a Drosophila tauopathy model
Source: Biol Open. 2026 Jul 28;15(7):bio062644. doi: 10.1242/bio.062644 (PMC13474566; doi:10.1242/bio.062644)
Supplement: Supplementary file 2 [file biolopen-15-062644_review_history.pdf]

Context-dependent toxicity of human Tau isoforms in a Drosophila tauopathy model

Yelena Ivanova, Miguel Ramirez-Moreno, Jie Liu, Leila Abtahi, Baidong Wu, Amber S. Cooper, Zengbo Wang, Douglas W. Allan, Amritpal Mudher, Aaron Comeault and Lovesha Sivanantharajah

10.1242/bio.062644

Editor: Sandhya Koushika

Review timeline

Original submission:	21 April 2026
Editorial decision:	27 April 2026
First revision received:	16 June 2026
Accepted:	25 June 2026

Original submission

First decision letter

MS ID#: bio.062644

MS Title: Context-dependent toxicity of human Tau isoforms in aÂ DrosophilaÂ tauopathy model

Authors: Yelena Ivanova, Miguel Ramirez-Moreno, Jie Liu, Leila Abtahi, Baidong Wu, Amber S. Cooper, Zengbo Wang, Douglas W. Allan, Amritpal Mudher, Aaron Comeault and Lovesha Sivanantharajah

I have now reached a decision on the above manuscript.

The reviewer reports are shown at the bottom of this email.

As you will see, the reviewers gave favourable reports, but raised some critical points that will require amendments to your manuscript. I hope that you will be able to carry these out, because we would like to be able to accept your paper.

At this stage, we also ask you to ensure your manuscript complies with our formatting guidelines - please see our manuscript preparation guidelines for details. Provided you are able to fully address the referees' comments, we are positive about publication of your paper (we accept over 95% of revision submissions) and therefore hope you won't mind any extra work involved in reformatting your manuscript at this point.

Please upload both a 'clean' version of your Word file, along with a highlighted version clearly showing where you have made changes in the revised manuscript. Please avoid using 'Track changes' in Word files as these are lost in PDF conversion.

I should be grateful if you would also provide a point-by-point response detailing how you have dealt with the points raised by the reviewers in the 'Response to Reviewers' box. Please attend to all of the reviewers' comments. If you do not agree with any of their criticisms or suggestions please explain clearly why this is so.

© 2026. Published by The Company of Biologists under the terms of the Creative Commons Attribution License (https://creativecommons.org/licenses/by/4.0/).

Reviewer 1

Comments for the author

Thank you for submitting your work.

Summary
This manuscript presents a useful new Drosophila resource for comparing all six human tau isoforms under matched transgenic conditions and applies it across multiple phenotypic contexts. The study is well motivated, the experiments are broad in scope, and the main conclusion that tau isoform toxicity is context dependent is interesting and potentially valuable to the field. The data are generally clear, and the paper is well written. My comments below are minor and mainly concern wording, clarification of experimental design, and presentation.
Minor comments
1. The manuscript is clearly written overall, but some statements in the Discussion would benefit from slightly more cautious wording.
2. In particular, the claim that resilience may represent a transient state in which "most or all neurons" eventually succumb appears broader than the data shown and should be softened.
3. Similarly, the statement that toxicity differences are not explained by hTau abundance and phosphorylation should be qualified, since only total tau and AT8-positive tau were assessed.
4. The wording around "equivalent expression" of the six isoforms should be refined to make clear that expression was broadly comparable at early time points, but not identical at later stages.
5. Please clarify more explicitly, in the main text and/or a summary table, which assays used adult-restricted expression, and which involved developmental expression, as this is important for interpreting context dependence.
6. The manuscript would benefit from a clearer distinction between "toxicity," "degeneration," and "dysfunction," since these terms are used across different assays with different biological meanings.
7. Please state explicitly whether all experiments were performed in males only and briefly acknowledge this as a limitation if applicable.
8. It would be helpful to clarify the rationale for using LacZ as the overexpression control.
9. For imaging experiments, please report sample structure more clearly, including the number of animals, VNSs, and measurements contributing to each quantified dataset.
10. Please indicate whether image acquisition and quantification were performed blinded to genotype.
11. Reporting effect sizes and confidence intervals alongside p values would improve interpretation, especially where conclusions rely on absence of significant differences.
12. Where possible, showing individual data points for quantified imaging data would improve transparency.
13. Figure 6 is a useful overview, but the legend should more clearly emphasize that it summarizes statistical groupings rather than effect magnitudes.
14. The Discussion of the VQIINK motif is interesting, but it should be framed clearly as a mechanistic hypothesis rather than a directly tested explanation in this study.
15. Because human tauopathies often involve mixtures of isoforms, the authors may wish to state this limitation more explicitly when discussing translational relevance.
16. Terminology should be checked for consistency, for example use of "VNC" versus "VNS."
17. Citation formatting should be standardized throughout the manuscript.
18. A final proofreading pass would help address minor formatting inconsistencies in the Methods and figure legends.

Reviewer 2

Comments for the author

I find the work to address an important question in tau neurobiology, and the high-resolution subcellular quantification within central nervous system hemilineages represents a notable technical achievement. However, several critical issues—undermine the findings. The authors should address the following points.
Comment 1:
The authors must detail the complete genetic provenance of the UAS-LacZ line in the methodology section; if the exact promoter strength or specific genomic insertion sites do not perfectly match

© 2026. Published by The Company of Biologists under the terms of the Creative Commons Attribution License (https://creativecommons.org/licenses/by/4.0/).

the VK00037 tau lines, the authors must provide supplementary qPCR data proving that the transcriptional and translational loads are mathematically equivalent, or formally concede this genetic discrepancy as a significant, interpretation-altering limitation in their experimental design.
Comment 2:
A major strength of the manuscript is the high-resolution subcellular quantification of both total human tau (hTau) and the specifically hyperphosphorylated AT8 epitope (Ser202/Thr205) within the targeted 19B and 6A central nervous system hemilineages, which the authors utilize to interrogate the mechanistic uncoupling of simple aggregate protein burden from the actual severity of the observed structural degeneration. However, this biochemical scrutiny is abandoned the moment the analysis shifts to the peripheral tissue assays, namely the engrailed-GAL4 driven posterior wing compartment and the ninaE.GMR-GAL4 driven visual system. The authors conclude that the 4R tau isoforms are generally more toxic than their 3R counterparts in these non-CNS environments, implicitly suggesting this observation is due to intrinsic structural differences inherent to the tau molecule, such as the additional secondary microtubule-binding repeat or the presence of the highly aggregative VQIINK hexapeptide motif. Yet, without any corresponding immunohistochemical or Western blot quantification of AT8 or alternative critical phospho-epitopes specifically extracted from the affected wing discs or eye imaginal structures, it remains scientifically possible that the apparent heightened toxicity of the 4R isoforms is merely a secondary consequence of tissue-specific kinase networks phosphorylating the 4R sequence much more efficiently than the 3R sequence in those exact, highly specialized peripheral locations. To substantiate the claim that the fundamental physical structure of the tau molecule itself interacts differently with the cellular environment to dictate overarching toxicity, the authors must supply supplementary biochemical or immunohistochemical data illustrating the actual phosphorylation status of the distinct tau isoforms within the wing and eye tissues, ensuring that the observed 3R vs 4R toxicity dichotomy is not simply an artifact of differential post-translational modification efficiency occurring uniquely in non-neuronal or specialized peripheral sensory environments.
Comment 3:
In the evaluation of tau-induced locomotor dysfunction, the authors deploy a repeated-measure two-way analysis of variance (ANOVA) to longitudinally track the negative geotaxis climbing performance of the OK371-GAL4 driven cohorts over a continuous, multi-week period. While the application of this specific statistical model is generally appropriate for mapping longitudinal behavioral datasets, the methodology section entirely omits critical information regarding the validation of the foundational mathematical assumptions required for repeated-measures ANOVA, most notably the assumption of sphericity, which dictates that the variances of the differences between all possible pairs of within-subject conditions are mathematically equal. Given the inherent, unavoidable auto-correlation strictly present in biological degradation over time—where the physical motor performance of a given fly at week 4 is dependent on its already degraded physical state at week 3—longitudinal Drosophila climbing data almost invariably violate the assumption of sphericity. The authors must explicitly state in the methods section whether Mauchly's test for sphericity was performed prior to analysis, and if a violation was indeed detected, they must confirm the application of an appropriate mathematical correction, such as the Greenhouse-Geisser or Huynh-Feldt epsilon adjustments to the degrees of freedom, ensuring that the critical P-values denoting the delayed emergence of the 2N4R versus 0N4R locomotor disparity are not the erroneous result of Type I error rates.
Comment 4:
The initial presentation of the longevity data in Figure 2A involves plotting the Kaplan-Meier survival curves for all seven distinct genetic cohorts—the six specific hTau isoforms alongside the beta-galactosidase control—simultaneously on a single, continuous two-dimensional axis system. This dense formatting choice results in graphical saturation, where the descending trajectories of the intermediate toxicity isoforms, specifically 1N4R and 2N3R, repeatedly cross over and overlap to such a degree that accurate visual discrimination of their exact temporal decay rates becomes difficult for the reader to independently verify. While the text briefly reports the statistical outcomes of the applied Cox proportional hazards model in the form of exact Hazard Ratios (HR), the manuscript requires a supplement to bridge the gap between the visual representation and the statistical outputs. The authors should construct a supplementary table detailing the exact median survival time, the maximum survival time, and the corresponding calculated 95% confidence intervals (expressed in precise days post-eclosion) for every individual analyzed genotype.
Comment 5:
The methodology relies upon the TARGET system, which necessitates raising the initial transgenic crosses at 18°C before physically transferring the newly eclosed adult flies into a significantly

© 2026. Published by The Company of Biologists under the terms of the Creative Commons Attribution License (https://creativecommons.org/licenses/by/4.0/).

warmer 29°C environment. This sudden 11-degree Celsius thermal upshift is not a biologically neutral event; it represents an environmental stressor that induces a robust endogenous heat shock response throughout the Drosophila organism, triggering vast transcriptomic alterations and mobilization of endogenous molecular chaperones, proteases, and stress-response kinase cascades. The manuscript would greatly benefit from a brief discussion in the text acknowledging this unavoidable intersection of thermal stress and acute proteotoxicity, clarifying whether the 18°C to 29°C shift might artificially prime the cellular autophagic machinery in a manner that either artificially buffers or synergistically exacerbates the early emergence of the isoform-specific phenotypic deficits observed in the initial days following eclosion.
Comment 6:
Throughout the analysis of the 19B and 6A hemilineages, the manuscript positions the AT8 monoclonal antibody as the singular proxy for the total cellular burden of "tau pathology," using its signal intensity to claim that structural differences in toxicity cannot be fully explained by phosphorylation states. It is imperative to refine this nomenclature within the text to accurately reflect the complex biochemical reality that the AT8 antibody is highly specific, recognizing only the phosphorylation events occurring explicitly at the paired Serine-202 and Threonine-205 residues. While Ser202/Thr205 hyperphosphorylation is a critical, early hallmark of paired helical filament initiation, the vast, diverse landscape of human tauopathies features complex hierarchical phosphorylation cascades involving numerous other critical epitopes—such as the AT100 site indicative of mature filamentous aggregation, the pSer262 site associated with profound microtubule detachment, or the PHF-1 site associated with end-stage neurofibrillary tangles. The authors must temper their declarative statements by acknowledging in the text that the lack of direct correlation between the AT8-positive signal and the severity of tissue degeneration does not in any way preclude the possibility that unmeasured phosphorylation events at other epitopes might predict the divergent isoform toxicity profiles observed across the targeted neuronal hemilineages.
Comment 7:
The quantification of protein expression via Western blot densitometry and the high-resolution measurement of subcellular corrected total cell fluorescence (CTCF) both rely entirely on the binding dynamics of a single, specific primary rabbit anti-hTau polyclonal antibody mixture (Agilent - DAKO, Cat. no. A002401-2). The manuscript operates on the assumption that this polyclonal reagent exhibits unvarying binding affinity across all six tau protein configurations, despite the structural variations inherent to alternative splicing, including the absence of the lengthy N-terminal inserts (0N vs 2N) and the presence or absence of the highly structured second microtubule-binding repeat (3R vs 4R). Because the premise of the "equivalent expression levels" argument hinges on the reliability of this single immunological tool, the authors must provide citations from previous literature, or submit supplementary biochemical data (such as recombinant protein dot-blot serial dilutions), proving that the DAKO polyclonal mixture detects the smaller 48 kDa 0N3R isoform with the same efficiency as the67 kDa 2N4R isoform, ruling out the possibility that perceived expression differences (or lack thereof) are merely artifacts of differential, sequence-specific epitope availability.
Comment 8:
The authors must insert a statement into the discussion section that frames the reported hierarchies of toxicity as a male-specific phenomena, while acknowledging the need for adequately powered follow-up investigations utilizing female cohorts to determine if these intricate isoform-specific interactions are genuinely conserved across sexes.
Comment 9:
The authors should revise the main text and all corresponding supplementary tables to ensure that exact, precise P-values are reported to at least three significant decimal places for every single statistical test executed throughout the study.

Reviewer's Responses to Questions

Experimental quality
Are experiments performed using appropriate methods that will answer the question (or test the hypothesis or support the observations) posed by the authors? Is the right tool used for the job?
If 'No', please indicate reasons in Comments for Author box below.
Reviewer #1:

- Yes

Reviewer #2:

© 2026. Published by The Company of Biologists under the terms of the Creative Commons Attribution License (https://creativecommons.org/licenses/by/4.0/).

- Yes

Were the data analyzed using appropriate statistical tests?
If 'No', please indicate reasons in Comments for Author box below.
Reviewer #1:
- Yes

Reviewer #2:
- Yes

Reproducibility
Were experiments performed using adequate number of biological replicates?
If 'No', please indicate reasons in Comments for Author box below.
Reviewer #1:
- Yes

Reviewer #2:
- Yes

Is there sufficient raw data to assess the rigor of the analysis?
If 'No', please indicate reasons in Comments for Author box below.
Reviewer #1:
- Yes

Reviewer #2:
- Yes

Does the methods section provide sufficient detail to permit reproducibility?
If 'No', please indicate reasons in Comments for Author box below.
Reviewer #1:
- Yes

Reviewer #2:
- Yes

Completeness
Are the manuscript's conclusions supported by the data?
If 'No', please indicate reasons in Comments for Author box below.
Reviewer #1:
- Yes

Reviewer #2:
- Yes

Scholarship
Do the authors cite and discuss the merits of data that would argue for and against their conclusion?
If 'No', please indicate reasons in Comments for Author box below.
Reviewer #1:
- Yes

Reviewer #2:
- Yes

© 2026. Published by The Company of Biologists under the terms of the Creative Commons Attribution License (https://creativecommons.org/licenses/by/4.0/).

Does the manuscript title & abstract accurately reflect the contents of the manuscript, without hyperbole?
If 'No', please indicate reasons in Comments for Author box below.
Reviewer #1:
- Yes

Reviewer #2:
- Yes

First revision

Author response to reviewers' comments

We appreciate the reviewers' constructive feedback on our manuscript. We have revised the manuscript to address the comments and suggestions, including clarifying methodological details, refining the wording of several interpretations, and adding additional supplementary information where appropriate. We believe these revisions have improved the clarity and balance of the manuscript, and we hope that it is now suitable for publication.

Reviewer comments are shown in normal text, with our point-by-point responses colour-coded in blue. Changes made in the revised manuscript are highlighted in yellow.

Reviewer 1:

R1-1: The manuscript is clearly written overall, but some statements in the Discussion would benefit from slightly more cautious wording.
R1-2: In particular, the claim that resilience may represent a transient state in which "most or all neurons" eventually succumb appears broader than the data shown and should be softened.

We have addressed these comments together as they relate to the same issue. We agree with the reviewer and have softened the relevant interpretation. The revised text describes the phenotype as an age-associated reduction in resilience, rather than implying that most or all neurons eventually succumb to tau toxicity.

Pg. 2, line 48:
"In selected neuronal populations, neurons classed as vulnerable showed early degeneration and continued decline, whereas resilient neurons degenerated only at the later time point. This suggests that, in some neurons, resilience may reflect early resistance to disease processes that is lost with age and exposure to age-related stressors."

R1-3: Similarly, the statement that toxicity differences are not explained by hTau abundance and phosphorylation should be qualified, since only total tau and AT8-positive tau were assessed.

We agree and have revised the manuscript to use more cautious and consistent wording around hTau expression, abundance, and phosphorylation. We have clarified in the Results section that the abundance measurements represent detectable total hTau signal, and that AT8-positive tau reflects phosphorylation at a specific epitope rather than the broader phosphorylation state of tau. These points are also now acknowledged as limitations in the Discussion section.

Pg. 11, line 455:
"Importantly, this pattern was not fully accounted for by detectable total hTau signal or AT8-positive tau levels alone. Rather than reflecting detectable tau levels or phosphorylation at a single disease-relevant epitope, it is possible that toxicity depends on how tau behaves within a given neuronal context: its subcellular localisation, conformational state, broader post-

© 2026. Published by The Company of Biologists under the terms of the Creative Commons Attribution License (https://creativecommons.org/licenses/by/4.0/).

translational modification profile at other disease-relevant phospho-sites, and interactions with cell-type-specific pathways."

R1-4: The wording around "equivalent expression" of the six isoforms should be refined to make clear that expression was broadly comparable at early time points, but not identical at later stages.

We have revised the wording to avoid implying equivalent expression of the six hTau isoforms across all experimental stages. The revised text now refers specifically to the early adult time point, where protein quantification detected no significant differences in hTau between the six transgenic lines.

Pg. 2, line 44:
"In young adults, hTau abundance did not differ significantly among lines, yet expression levels were sufficient to induce visible, isoform-specific phenotypes."

R1-5: Please clarify more explicitly, in the main text and/or a summary table, which assays used adult-restricted expression, and which involved developmental expression, as this is important for interpreting context dependence.

We have revised Figure 6 and its legend to clarify both points. We added an expression window column indicating whether each assay used adult-restricted or developmental hTau expression.

Pg. 21, line 859:
"**Fig. 6**. Summary of results: context-dependent toxicity of the hTau isoforms. Each cell is coloured according to the post-hoc statistical groupings within that assay ($p < 0.05$), providing a categorical summary of relative toxicity rather than a proportional measure of effect magnitude to gauge general isoform-specific differences. Colours were assigned based on the assay metric, including reduced lifespan, increased degeneration, higher phenotypic score, or decreased neuronal area. Red indicates the most toxic group for an assay, and blue denotes no significant difference from the β-galactosidase (LacZ) control. Where a cell contains two colours, the isoform toxicity is not significantly different from either colour group, although these groups differ from each other. No continuous scoring system was used, and no numerical values were assigned to the colours. The expression window indicates whether hTau expression was adult-restricted using the TARGET system, driven by developmental and adult-expressing GAL4 drivers (GMR, en), or confined to post-mitotic neurons using split-GAL4 hemilineage drivers."

R1-6: The manuscript would benefit from a clearer distinction between "toxicity," "degeneration," and "dysfunction," since these terms are used across different assays with different biological meanings.

We agree and have revised the manuscript to use these terms more consistently. We now use "toxicity" as a broader term for adverse effects of hTau expression across assays/tissues, while referring more specifically to "degeneration" for structural readouts such as wing/eye morphology, neuronal area, and presynaptic area, and "dysfunction" for behavioural impairment in the negative geotaxis assay. We have also revised the relevant text in Results and Discussion to make the assay-specific meaning clearer.

R1-7: Please state explicitly whether all experiments were performed in males only and briefly acknowledge this as a limitation if applicable.

We thank the reviewer for raising this point. We have clarified the sex used for each assay in the Methods section. All experiments except those described in the "tau isoforms and selective vulnerability" section, corresponding to the 6A and 19B hemilineage immunohistochemistry experiments, were performed in males. The 6A and 19B hemilineage experiments were performed in females. We have also added a limitation in the Discussion noting that sex-dependent effects were not systematically assessed in this study.

Pg. 12, line 488:
"Finally, all assays were performed in males, with the exception of 19B and 6A VNS immunohistochemistry experiments, which were performed in females to maintain consistency with

© 2026. Published by The Company of Biologists under the terms of the Creative Commons Attribution License (https://creativecommons.org/licenses/by/4.0/).

previously published studies (Sivanantharajah et al., 2025). Potential sex-dependent modulation of isoform-specific toxicity was not evaluated in this study."

R1-8: It would be helpful to clarify the rationale for using LacZ as the overexpression control.

We thank the reviewer for raising this point and have clarified the rationale for using LacZ as the control in the text. LacZ was used as a non-tau UAS transgene control to distinguish hTau-associated phenotypes from effects related to GAL4/UAS-driven expression of an exogenous protein (i.e., protein over-expression of a non-toxic protein alone). We have also added references to support the use of LacZ as a control in Drosophila studies.

Pg. 14, line 564:
"LacZ was used as a non-tau UAS transgene control to distinguish hTau-associated phenotypes from effects related to GAL4/UAS-driven transgene expression alone (Martin-Pena et al., 2018), (Xu et al., 2015)."

R1-9: For imaging experiments, please report sample structure more clearly, including the number of animals, VNSs, and measurements contributing to each quantified dataset.

We thank the reviewer for these suggestions. Individual data points are shown for the quantified imaging datasets, and we have revised the figure legends to make this explicit. We have also clarified the sample structure for each dataset, including the number of animals/VNSs analysed (Fig. 4 and Fig. 5) and, for the presynaptic area analysis, the number of measurements obtained per VNS (Fig. 5C). For this analysis, VNS identity was included as a random effect in the linear mixed-effects model.

Pg. 20, line 837 and pg. 21, line 847:
"Individual points represent single VNSs from individual animals…"

Pg. 21, line 853:
"Individual points represent quantified presynaptic measurements from the same VNSs analysed in **B**, with two measurements obtained per VNS. Statistical analysis was performed using a linear mixed-effects model with VNS identity included as a random effect."

R1-10: Please indicate whether image acquisition and quantification were performed blinded to genotype.

We thank the reviewer for raising this point. Image acquisition and quantification were not performed blinded to genotype. We have now stated this explicitly in the Methods section and acknowledge it as a limitation of the imaging analyses.

Pg. 15, line 609:
"Images used for signal quantification were obtained at 40x oil magnification using identical image acquisition parameters. Image acquisition and quantification were not performed blinded to genotype."

R1-11: Reporting effect sizes and confidence intervals alongside p values would improve interpretation, especially where conclusions rely on absence of significant differences.

We agree that effect estimates and confidence intervals are useful for interpreting results, particularly for measurements where no statistically significant differences were detected. We have therefore generated a supplementary table reporting effect estimates, 95% confidence intervals, and p-values for the hTau and AT8-positive tau measurements (Table S2). This table is cited in the relevant Results sections.

R1-12: Where possible, showing individual data points for quantified imaging data would improve transparency.

We have addressed this above in R1-9.

© 2026. Published by The Company of Biologists under the terms of the Creative Commons Attribution License (https://creativecommons.org/licenses/by/4.0/).

R1-13: Figure 6 is a useful overview, but the legend should more clearly emphasize that it summarizes statistical groupings rather than effect magnitudes.

We changed the legend to state more explicitly that the colours summarise post-hoc statistical groupings within each assay and should not be interpreted as proportional effect magnitudes across assays.

R1-14: The Discussion of the VQIINK motif is interesting, but it should be framed clearly as a mechanistic hypothesis rather than a directly tested explanation in this study.

We agree and have revised this section to make the interpretation of VQIINK more cautious. VQIINK is now presented as a possible contributor to the stronger 4R-associated phenotypes, rather than as a mechanism directly demonstrated by the present data.

R1-15: Because human tauopathies often involve mixtures of isoforms, the authors may wish to state this limitation more explicitly when discussing translational relevance.

We have revised the text to make this limitation clearer, noting that isolated isoform expression in *Drosophila* does not fully reflect human tauopathies in which multiple tau isoforms often coexist.

As these two comments refer to the same Discussion paragraph, the revised text addressing both **R1-14** and **R1-15** is provided below:

Pg. 9, line 364:

"The higher toxicity of 4R isoforms aligns well with the isoform imbalance observed in human tauopathies. Primary tauopathies, such as PSP, CBD, and many others are characterised by the pathological accumulation of 4R tau, whereas 3R-dominant pathology is rare (Murray et al., 2014, Mailliot et al., 1998). While this supports the relevance of isoform-specific toxicity, isolated isoform expression in *Drosophila* does not fully capture human tauopathies, where multiple tau isoforms often coexist and undergo disease stage-dependent biochemical changes. One possible explanation for the 3R-4R differences observed here is the presence of an additional hexapeptide aggregation motif, VQIINK, in the R2 repeat of 4R tau (von Bergen et al., 2001). VQIINK can form highly stable steric zippers leading to strong self-aggregation capacity (Seidler et al., 2018). Although VQIVYK is essential for filament formation, VQIINK deletion has been linked to reduced tau assembly, raising the possibility that VQIINK may contribute to enhanced 4R toxicity (Zhang et al., 2019). This remains a mechanistic hypothesis, however, and was not directly tested in the present study."

R1-16: Terminology should be checked for consistency, for example use of "VNC" versus "VNS."
R1-17: Citation formatting should be standardized throughout the manuscript.
R1-18: A final proofreading pass would help address minor formatting inconsistencies in the Methods and figure legends.

We thank the reviewer for these comments. We have addressed these points during a final proofreading of the manuscript. We have standardised the anatomical terminology and now use "VNS" throughout, checked citation formatting for consistency, and corrected minor formatting issues in the Methods and figure legends.

Reviewer 2:

R2-1: The authors must detail the complete genetic provenance of the UAS-LacZ line in the methodology section; if the exact promoter strength or specific genomic insertion sites do not perfectly match the VK00037 tau lines, the authors must provide supplementary qPCR data proving that the transcriptional and translational loads are mathematically equivalent, or formally concede this genetic discrepancy as a significant, interpretation-altering limitation in their experimental design.

We thank the reviewer for raising this point. We have clarified the identity and role of the UAS-LacZ control in the Methods section (see above, R1-8) and provide the stock information in Table S1. LacZ was used as a non-tau UAS transgene control to distinguish hTau-associated phenotypes

© 2026. Published by The Company of Biologists under the terms of the Creative Commons Attribution License (https://creativecommons.org/licenses/by/4.0/).

from effects related to GAL4/UAS-driven expression of any exogenous protein, even one that is not toxic. We also clarify that this control is not genetically equivalent to VK00037 10xUAS-hTau isoform lines and should not be interpreted as an insertion site-, UAS copy-number-, or expression load-matched control. Accordingly, comparisons with LacZ are interpreted as comparisons to a non-tau transgene control, whereas the main isoform-specific conclusions are based on comparisons among the six matched hTau lines.

Pg. 14, line 563:

"Elav-GAL4, tubGal80^{ts} flies were crossed to each of the six UAS-hTau isoform lines and UAS-lacZ (BDSC #8529; Table S1). LacZ was used as a non-tau UAS transgene control to distinguish hTau-associated phenotypes from effects related to GAL4/UAS-driven transgene expression alone (Martin-Pena et al., 2018), (Xu et al., 2015)."

R2-2: A major strength of the manuscript is the high-resolution subcellular quantification of both total human tau (hTau) and the specifically hyperphosphorylated AT8 epitope (Ser202/Thr205) within the targeted 19B and 6A central nervous system hemilineages, which the authors utilize to interrogate the mechanistic uncoupling of simple aggregate protein burden from the actual severity of the observed structural degeneration. However, this biochemical scrutiny is abandoned the moment the analysis shifts to the peripheral tissue assays, namely the engrailed-GAL4 driven posterior wing compartment and the ninaE.GMR-GAL4 driven visual system. The authors conclude that the 4R tau isoforms are generally more toxic than their 3R counterparts in these non-CNS environments, implicitly suggesting this observation is due to intrinsic structural differences inherent to the tau molecule, such as the additional secondary microtubule-binding repeat or the presence of the highly aggregative VQIINK hexapeptide motif. Yet, without any corresponding immunohistochemical or Western blot quantification of AT8 or alternative critical phospho-epitopes specifically extracted from the affected wing discs or eye imaginal structures, it remains scientifically possible that the apparent heightened toxicity of the 4R isoforms is merely a secondary consequence of tissue-specific kinase networks phosphorylating the 4R sequence much more efficiently than the 3R sequence in those exact, highly specialized peripheral locations. To substantiate the claim that the fundamental physical structure of the tau molecule itself interacts differently with the cellular environment to dictate overarching toxicity, the authors must supply supplementary biochemical or immunohistochemical data illustrating the actual phosphorylation status of the distinct tau isoforms within the wing and eye tissues, ensuring that the observed 3R vs 4R toxicity dichotomy is not simply an artifact of differential post-translational modification efficiency occurring uniquely in non-neuronal or specialized peripheral sensory environments.

We agree that the wing and eye phenotypes should not be overinterpreted as direct evidence for a specific biochemical mechanism. Previous work in *Drosophila* has shown that hTau can undergo cell-type-specific processing, including differences in phosphorylation and stability, likely reflecting local regulation of tau-associated kinases and phosphatases (Grammenoudi et al., 2006). Therefore, tissue-specific biochemical regulation could contribute to the wing and eye phenotypes observed in the study. However, higher toxicity of 4R isoforms was observed across multiple assays and tissues, suggesting that tissue-specific post-translational modification alone is unlikely to fully account for the 3R/4R toxicity pattern. We have revised the Discussion section to acknowledge that wing and eye assays were not accompanied by tissue-specific biochemical analysis, while also framing the broader toxicity pattern as likely reflecting interactions between isoform-intrinsic factors and the cellular environment. We have also softened the VQIINK/R2 discussion so that it is framed as a mechanistic hypothesis rather than a directly tested explanation.

Grammenoudi S, Kosmidis S, Skoulakis EM. Cell type-specific processing of human Tau proteins in Drosophila. FEBS Lett. 2006;580(19):4602-6.

Pg. 12, line 483:

"Third, the wing and eye assays provide phenotypic readouts of isoform-dependent toxicity; however, they were not accompanied by tissue-specific biochemical analysis here. While tissue-specific modification of tau may contribute to these phenotypes, the consistent emergence of stronger 4R-associated toxicity across multiple assays and tissue types suggests that these factors are unlikely to fully account for the overall 3R/4R pattern."

© 2026. Published by The Company of Biologists under the terms of the Creative Commons Attribution License (https://creativecommons.org/licenses/by/4.0/).

R2-3: In the evaluation of tau-induced locomotor dysfunction, the authors deploy a repeated-measure two-way analysis of variance (ANOVA) to longitudinally track the negative geotaxis climbing performance of the OK371-GAL4 driven cohorts over a continuous, multi-week period. While the application of this specific statistical model is generally appropriate for mapping longitudinal behavioral datasets, the methodology section entirely omits critical information regarding the validation of the foundational mathematical assumptions required for repeated-measures ANOVA, most notably the assumption of sphericity, which dictates that the variances of the differences between all possible pairs of within-subject conditions are mathematically equal. Given the inherent, unavoidable auto-correlation strictly present in biological degradation over time—where the physical motor performance of a given fly at week 4 is dependent on its already degraded physical state at week 3—longitudinal Drosophila climbing data almost invariably violate the assumption of sphericity. The authors must explicitly state in the methods section whether Mauchly's test for sphericity was performed prior to analysis, and if a violation was indeed detected, they must confirm the application of an appropriate mathematical correction, such as the Greenhouse-Geisser or Huynh-Feldt epsilon adjustments to the degrees of freedom, ensuring that the critical P-values denoting the delayed emergence of the 2N4R versus 0N4R locomotor disparity are not the erroneous result of Type I error rates.

We thank the reviewer for raising this point. We have clarified in the Methods section that Mauchly's test was not used as a decision point for the climbing analysis. Given the repeated longitudinal structure of the dataset, the repeated-measures two-way ANOVA was performed in Prism with the Geisser-Greenhouse correction applied to account for potential violations of sphericity. The reported "time x hTau isoform" interaction is based on the corrected degrees of freedom ($p = 0.0395$; Geisser-Greenhouse $\varepsilon = 0.5828$). Post hoc comparisons at individual time points were interpreted within this corrected repeated-measures framework.

Pg. 15, line 629:

"Repeated measures two-way ANOVA with Tukey-corrected post-hoc tests was used to analyse the effects of isoforms on climbing ability (Table S5). Due to the longitudinal structure of the climbing dataset, the Geisser-Greenhouse correction was applied to account for potential violations of sphericity."

R2-4: The initial presentation of the longevity data in Figure 2A involves plotting the Kaplan-Meier survival curves for all seven distinct genetic cohorts—the six specific hTau isoforms alongside the beta-galactosidase control—simultaneously on a single, continuous two-dimensional axis system. This dense formatting choice results in graphical saturation, where the descending trajectories of the intermediate toxicity isoforms, specifically 1N4R and 2N3R, repeatedly cross over and overlap to such a degree that accurate visual discrimination of their exact temporal decay rates becomes difficult for the reader to independently verify. While the text briefly reports the statistical outcomes of the applied Cox proportional hazards model in the form of exact Hazard Ratios (HR), the manuscript requires a supplement to bridge the gap between the visual representation and the statistical outputs. The authors should construct a supplementary table detailing the exact median survival time, the maximum survival time, and the corresponding calculated 95% confidence intervals (expressed in precise days post-eclosion) for every individual analyzed genotype.

We thank the reviewer for this helpful suggestion. We have revised Figure 2A to reduce graphical saturation and improve visual discrimination between genotypes. We also added a new supplementary table reporting the median survival time with 95% confidence intervals and the maximum observed survival time for each genotype (Table S4).

R2-5: The methodology relies upon the TARGET system, which necessitates raising the initial transgenic crosses at 18°C before physically transferring the newly eclosed adult flies into a significantly warmer 29°C environment. This sudden 11-degree Celsius thermal upshift is not a biologically neutral event; it represents an environmental stressor that induces a robust endogenous heat shock response throughout the Drosophila organism, triggering vast transcriptomic alterations and mobilization of endogenous molecular chaperones, proteases, and stress-response kinase cascades. The manuscript would greatly benefit from a brief discussion in the text acknowledging this unavoidable intersection of thermal stress and acute proteotoxicity, clarifying whether the 18°C to 29°C shift might artificially prime the cellular autophagic machinery in a

© 2026. Published by The Company of Biologists under the terms of the Creative Commons Attribution License (https://creativecommons.org/licenses/by/4.0/).

manner that either artificially buffers or synergistically exacerbates the early emergence of the isoform-specific phenotypic deficits observed in the initial days following eclosion.

We agree that the temperature shift required for TARGET-mediated adult induction is an important experimental consideration. We have added a statement in the Discussion section acknowledging that TARGET-based adult phenotypes should be interpreted in the context of the shared 18°C-to-29°C temperature shift paradigm, which may influence stress response and proteostasis pathways. Because all genotypes, including LacZ controls, were exposed to the same temperature shift, this is unlikely to account for isoform-specific differences by itself. We have also noted that previous TARGET-based tau work did not find adult induction of 4R hTau alone to be sufficient to produce detectable degenerative phenotypes, supporting the view that the toxicity observed in our study depends on robust hTau expression and isoform-specific properties rather than the temperature shift alone.

Pg. 9, line 353:

“Adult-restricted expression using the TARGET system requires transfer from 18°C to 29°C, so TARGET-based adult phenotypes should be interpreted in the context of a shared temperature-shift paradigm that may influence stress response and proteostasis pathways (Bettencourt et al., 2008), (Donovan and Marr, 2016). Because this shift was applied across all genotypes, including controls, it is unlikely to account for isoform-specific differences by itself. Importantly, previous TARGET-based work has shown that adult induction of 4R hTau expression does not necessarily produce detectable degenerative phenotypes (Malmanche et al., 2017), suggesting that the emergence of toxicity depends on robust hTau expression together with isoform-specific properties, rather than heat-shock priming alone.”

R2-6: Throughout the analysis of the 19B and 6A hemilineages, the manuscript positions the AT8 monoclonal antibody as the singular proxy for the total cellular burden of "tau pathology," using its signal intensity to claim that structural differences in toxicity cannot be fully explained by phosphorylation states. It is imperative to refine this nomenclature within the text to accurately reflect the complex biochemical reality that the AT8 antibody is highly specific, recognizing only the phosphorylation events occurring explicitly at the paired Serine-202 and Threonine-205 residues. While Ser202/Thr205 hyperphosphorylation is a critical, early hallmark of paired helical filament initiation, the vast, diverse landscape of human tauopathies features complex hierarchical phosphorylation cascades involving numerous other critical epitopes—such as the AT100 site indicative of mature filamentous aggregation, the pSer262 site associated with profound microtubule detachment, or the PHF-1 site associated with end-stage neurofibrillary tangles. The authors must temper their declarative statements by acknowledging in the text that the lack of direct correlation between the AT8-positive signal and the severity of tissue degeneration does not in any way preclude the possibility that unmeasured phosphorylation events at other epitopes might predict the divergent isoform toxicity profiles observed across the targeted neuronal hemilineages.

We agree that AT8 should be described more precisely. AT8 provides a widely used readout of tau pathology, but it detects phosphorylation at Ser202/Thr205 and should not be interpreted as capturing the full phosphorylation landscape of tau. We have revised the manuscript to refer to the “AT8-positive tau” where appropriate and to avoid broader statements implying that AT8 signal reflects overall tau phosphorylation state. We have also added a limitation noting that phosphorylation at other sites and other post-translational modifications may contribute to isoform-specific toxicity.

Pg. 12, line 478:

“Second, AT8, which is a widely used marker of disease-associated tau, was used to assess phosphorylation at Ser202/Thr205. However, AT8 represents one disease-relevant phospho-epitope, and it is possible that phosphorylation at other sites or other post-translational modifications not explored here may also contribute to isoform-specific toxicity.”

R2-7: The quantification of protein expression via Western blot densitometry and the high-resolution measurement of subcellular corrected total cell fluorescence (CTCF) both rely entirely on the binding dynamics of a single, specific primary rabbit anti-hTau polyclonal antibody mixture (Agilent - DAKO, Cat. no. A002401-2). The manuscript operates on the assumption that this polyclonal reagent exhibits unvarying binding affinity across all six tau protein configurations,

© 2026. Published by The Company of Biologists under the terms of the Creative Commons Attribution License (https://creativecommons.org/licenses/by/4.0/).

despite the structural variations inherent to alternative splicing, including the absence of the lengthy N-terminal inserts (0N vs 2N) and the presence or absence of the highly structured second microtubule-binding repeat (3R vs 4R). Because the premise of the "equivalent expression levels" argument hinges on the reliability of this single immunological tool, the authors must provide citations from previous literature, or submit supplementary biochemical data (such as recombinant protein dot-blot serial dilutions), proving that the DAKO polyclonal mixture detects the smaller 48 kDa 0N3R isoform with the same efficiency as the67 kDa 2N4R isoform, ruling out the possibility that perceived expression differences (or lack thereof) are merely artifacts of differential, sequence-specific epitope availability.

We thank the reviewer for raising this important point. We agree that antibody-based quantification cannot by itself prove identical absolute abundance across all six hTau isoforms. The reported recognition region for DAKO/Agilent (A0024) is within amino acids 243-441 of human tau, which does not overlap with the alternatively spliced N-terminal inserts that distinguish 0N, 1N, and 2N isoforms (Meredith et al., 2013), (Hochgrafe et al., 2013). However, because this region includes sequences affected by 3R/4R splicing, identical detection efficiency across all six isoforms cannot be assumed. We have therefore clarified that western blot densitometry and CTCF measurements represent detectable hTau signal using this reagent, rather than definitive measures of absolute isoform abundance. We have also revised wording around isoform expression in the abstract and added a limitation in the discussion noting that isoform-dependent differences in epitope availability or antibody-binding efficiency cannot be fully excluded.

Hochgrafe, K., Sydow, A. & Mandelkow, E. M. (2013). Regulatable transgenic mouse models of Alzheimer disease: onset, reversibility and spreading of Tau pathology. *FEBS J*, 280, 4371-81.
Meredith, J. E., Jr., Sankaranarayanan, S., Guss, V., Lanzetti, A. J., Berisha, F., Neely, R. J., Slemmon, J. R., Portelius, E., Zetterberg, H., Blennow, K., et al. (2013). Characterization of novel CSF Tau and ptau biomarkers for Alzheimer's disease. *PLoS One*, 8, e76523.

Pg. 12, line 474:
"Some limitations should be considered when interpreting these findings. First, total hTau measurements relied on the DAKO/Agilent polyclonal anti-tau antibody and therefore represent detectable protein signal with this reagent rather than definitive measures of absolute isoform abundance. Isoform-dependent differences in epitope availability or antibody-binding efficiency cannot be fully excluded."

R2-8: The authors must insert a statement into the discussion section that frames the reported hierarchies of toxicity as a male-specific phenomena, while acknowledging the need for adequately powered follow-up investigations utilizing female cohorts to determine if these intricate isoform-specific interactions are genuinely conserved across sexes.

We thank the reviewer for raising this point. We agree that the sex of the animals should be stated clearly and that potential sex-dependent effects should be acknowledged. We have clarified in the Methods section that most assays were performed in males, whereas the 19B and 6A VNS immunohistochemistry experiments were performed in females. We have also added a limitation in the Discussion section noting that potential sex-dependent modulation of isoform-specific toxicity was not systematically investigated.

Pg. 12, line 488:
"Finally, all assays were performed in males, with the exception of 19B and 6A VNS immunohistochemistry experiments, which were performed in females to maintain consistency with previously published studies (Sivanantharajah et al., 2025). Potential sex-dependent modulation of isoform-specific toxicity was not evaluated in this study."

R2-9: The authors should revise the main text and all corresponding supplementary tables to ensure that exact, precise P-values are reported to at least three significant decimal places for every single statistical test executed throughout the study.

We have revised the main text and supplementary tables to report exact p-values to at least three decimal places wherever available. Values below the reporting threshold of the statistical software

© 2026. Published by The Company of Biologists under the terms of the Creative Commons Attribution License (https://creativecommons.org/licenses/by/4.0/).

are reported as $p < 0.001$ or $p < 0.0001$, as appropriate, and p-value formatting has been standardised throughout.

Second decision letter

MS ID#: bio.062644R1

MS Title: Context-dependent toxicity of human Tau isoforms in aÂ DrosophilaÂ tauopathy model

Authors: Yelena Ivanova, Miguel Ramirez-Moreno, Jie Liu, Leila Abtahi, Baidong Wu, Amber S. Cooper, Zengbo Wang, Douglas W. Allan, Amritpal Mudher, Aaron Comeault and Lovesha Sivanantharajah

I am happy to tell you that your manuscript has been accepted for publication in Biology Open, pending our standard publication integrity checks. It was accepted on 25th June 2026.

Reviewer 1

Comments for the author

I thank the authors for their careful and constructive revision of the manuscript. In my view, the authors have addressed the reviewers' concerns thoroughly and appropriately. The revised manuscript now uses more cautious language when interpreting neuronal resilience and tau toxicity, clarifies the distinction between toxicity, degeneration, and dysfunction, and better explains the experimental contexts in which different assays were performed.

I also appreciate the added methodological clarifications, including the rationale for the LacZ control, the sex of animals used in different experiments, the sample structure for imaging analyses, and the acknowledgement that image acquisition and quantification were not blinded to genotype. The inclusion of additional supplementary information, including effect estimates, confidence intervals, survival summaries, and corrected statistical reporting, improves the transparency and interpretability of the work.

Importantly, the authors have appropriately tempered mechanistic claims regarding AT8-positive tau, VQIINK, tissue-specific phosphorylation, and isoform-intrinsic toxicity. These revisions make the Discussion more balanced and better aligned with the data presented.

Overall, I think the authors have done a good job addressing the comments, and the manuscript is now clearer, more rigorous, and more appropriately framed. I support acceptance of the manuscript.

Reviewer 2

Comments for the author

Thanks for addressing the comments.

Reviewer's Responses to Questions

Experimental quality
Are experiments performed using appropriate methods that will answer the question (or test the hypothesis or support the observations) posed by the authors? Is the right tool used for the job?
If 'No', please indicate reasons in Comments for Author box below.
Reviewer #1:

- Yes

© 2026. Published by The Company of Biologists under the terms of the Creative Commons Attribution License (https://creativecommons.org/licenses/by/4.0/).

Reviewer #2:

- Yes

Were the data analyzed using appropriate statistical tests?
If 'No', please indicate reasons in Comments for Author box below.
Reviewer #1:

- Yes

Reviewer #2:

- Yes

Reproducibility
Were experiments performed using adequate number of biological replicates?
If 'No', please indicate reasons in Comments for Author box below.
Reviewer #1:

- Yes

Reviewer #2:

- Yes

Is there sufficient raw data to assess the rigor of the analysis?
If 'No', please indicate reasons in Comments for Author box below.
Reviewer #1:

- Yes

Reviewer #2:

- Yes

Does the methods section provide sufficient detail to permit reproducibility?
If 'No', please indicate reasons in Comments for Author box below.
Reviewer #1:

- Yes

Reviewer #2:

- Yes

Completeness
Are the manuscript's conclusions supported by the data?
If 'No', please indicate reasons in Comments for Author box below.
Reviewer #1:

- Yes

Reviewer #2:

- Yes

Scholarship
Do the authors cite and discuss the merits of data that would argue for and against their conclusion?
If 'No', please indicate reasons in Comments for Author box below.
Reviewer #1:

- Yes

Reviewer #2:

© 2026. Published by The Company of Biologists under the terms of the Creative Commons Attribution License (https://creativecommons.org/licenses/by/4.0/).

- Yes

Does the manuscript title & abstract accurately reflect the contents of the manuscript, without hyperbole?
If 'No', please indicate reasons in Comments for Author box below.
Reviewer #1:

- Yes

Reviewer #2:

- Yes

© 2026. Published by The Company of Biologists under the terms of the Creative Commons Attribution License (https://creativecommons.org/licenses/by/4.0/).
